# Comparative analyses of gene networks mediating cancer metastatic potentials across lineage types

**DOI:** 10.1093/bib/bbae357

**Published:** 2024-07-22

**Authors:** Sheng Wang, Emily K Stroup, Ting-You Wang, Rendong Yang, Zhe Ji

**Affiliations:** Department of Biomedical Engineering, McCormick School of Engineering, Northwestern University, 2145 Sheridan Road, Evanston, IL 60628, United States; Department of Pharmacology, Feinberg School of Medicine, Northwestern University, 303 E Superior Street, Chicago, IL 60611, United States; Department of Urology, Feinberg School of Medicine, Northwestern University, 303 E Superior Street, Chicago, IL 60611, United States; Department of Urology, Feinberg School of Medicine, Northwestern University, 303 E Superior Street, Chicago, IL 60611, United States; Department of Biomedical Engineering, McCormick School of Engineering, Northwestern University, 2145 Sheridan Road, Evanston, IL 60628, United States; Department of Pharmacology, Feinberg School of Medicine, Northwestern University, 303 E Superior Street, Chicago, IL 60611, United States

**Keywords:** cancer metastasis, gene network, lineage types, drug response

## Abstract

Studies have identified genes and molecular pathways regulating cancer metastasis. However, it remains largely unknown whether metastatic potentials of cancer cells from different lineage types are driven by the same or different gene networks. Here, we aim to address this question through integrative analyses of 493 human cancer cells’ transcriptomic profiles and their metastatic potentials *in vivo*. Using an unsupervised approach and considering both gene coexpression and protein–protein interaction networks, we identify different gene networks associated with various biological pathways (i.e. inflammation, cell cycle, and RNA translation), the expression of which are correlated with metastatic potentials across subsets of lineage types. By developing a regularized random forest regression model, we show that the combination of the gene module features expressed in the native cancer cells can predict their metastatic potentials with an overall Pearson correlation coefficient of 0.90. By analyzing transcriptomic profile data from cancer patients, we show that these networks are conserved *in vivo* and contribute to cancer aggressiveness. The intrinsic expression levels of these networks are correlated with drug sensitivity. Altogether, our study provides novel comparative insights into cancer cells’ intrinsic gene networks mediating metastatic potentials across different lineage types, and our results can potentially be useful for designing personalized treatments for metastatic cancers.

## Introduction

Cancer metastasis is the major cause of patient death, and substantial research efforts have been devoted to dissecting molecular mechanisms driving the metastasis [1, 2]. The molecular pathways promoting cancer progression were summarized as 10 hallmarks of cancers, such as “sustaining proliferative signaling”, “resisting cell death”, and “inducing angiogenesis” [3]. The remodeling of each pathway can contribute to increased malignancy and metastatic potential of cancers.

Human cancer cell lines are valuable tools for the discovery of oncogenic mechanisms and therapeutic approaches [4, 5]. The expression of intrinsic gene regulatory networks in cancer cells determines their metastatic potentials *in vivo*. Oncogenic mechanisms can be revealed through molecular comparisons between metastatic versus non-metastatic cells [6, 7]. Most studies of metastasis have focused on a few cancer cell lines from a lineage type for characterization. However, it remains mostly unknown whether the pathways learned from one lineage can be applied to others. Especially, there is a lack of systematic comparison of molecular pathways mediating cancer metastasis across different lineage types. Identifying common metastasis-driving modules across lineage types can lead to novel cancer subtyping and associated therapeutic approaches. One bottleneck toward this issue is the experimental cost and labor-intensive work to measure the metastatic potentials of cancer cells *in vivo*.

Recently, a study named MetMap leveraged the single-cell barcoding and high-throughput sequencing method and measured the metastatic potentials of 493 cancer cells from 21 lineage types [8]. The study engineered each cell line to express a unique sequence barcode and green fluorescent protein/mCherry for cell sorting. Five weeks after they injected the pooled cells into the left ventricle of mice, the human cancer cells were sorted from five organs (brain, lung, liver, kidney, and bone). The metastatic potentials were quantified based on barcode abundances measured by RNA sequencing. These cells were also characterized by the Cancer Cell Line Encyclopedia (CCLE) project with transcriptomic profiling data [9]. Although the study used the MetMap dataset to examine the genetic mutations linked to cancer metastasis and showed that the transcription factor SREBF1 mediating the lipid synthesis is a regulator of breast-to-brain metastasis, they have not performed systematic comparative analyses of transcriptomic differences between metastatic versus non-metastatic cancer cells. Here, we aimed to leverage this unique dataset to examine the molecular pathways mediating metastasis across cell lineage types.

**Figure 1:**
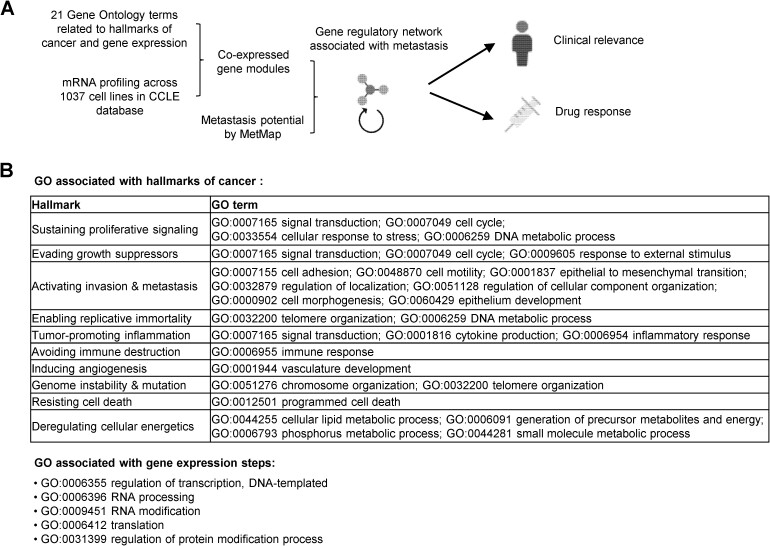
Overview of the experimental design. (A) Diagram showing data analysis steps. (B) 28 selected Gene Ontology (GO) terms related to hallmarks of cancer and steps of gene expression.

The gene coexpression network has been commonly used to identify regulatory pathways underlying a biological process [10–13]. Although cancer cells from different lineages are heterogeneous with cell type–specific regulation, some oncogenic pathways can be conserved. For example, we previously showed that genes in an inflammatory network mediated by the joint action of NF-*k*b, STAT3, and AP-1 transcription factors are coexpressed and the regulatory module is commonly active in many cancer types [14]. A study performed the integrated analyses of transcriptomic and patient survival data from the Cancer Genome Atlas (TCGA) and showed that cancer prognostic genes tend to form coexpressed modules showing cross-tumor conservation [15]. Here, to examine the gene networks mediating cancer metastasis, we aimed to obtain coexpressed gene modules in oncogenesis-related biological processes, identify module features predicting cancer cells’ metastatic potentials, and compare them across lineage types (Fig. 1A).

## Methods

### Identifying the coexpressed gene modules

We downloaded transcriptomic profiling data of 1037 cancer cell lines measured by microarray and cell type annotations from the CCLE database (https://portals.broadinstitute.org/ccle/) [9]. The relative expression level for gene *i* of cell line *j* was calculated as


(1) \begin{equation*} {E}_{ij}={R}_{ij}-\mathrm{Median}\left({R}_{i1},{R}_{i2},\dots{R}_{in}\right), \end{equation*}

where *R_ij_* is the Robust Multi-Array Average–normalized expression value [16] for gene *i* in cell line *j*, and *n* is the total number of cell lines included in the analyses. We removed lowly expressed genes and only protein-coding genes with expression levels ${R}_{ij}$ > 3 in at least 50% of cell lines were included in the following analyses.

For a GO term of interest, we retrieved associated genes with the R library “org.Hs.eg.db” (version 3.11.4). Then, we used the following unsupervised analysis steps using the relative gene expression levels to obtain the coexpressed modules named subGOs. First, we calculated the Spearman correlation coefficients of pairwise genes in a GO term across cancer cell lines, performed hierarchical clustering of the coefficient values, and calculated the Euclidean distances. Second, the correlation coefficients were randomly shuffled, and 5% false discovery rate (FDR) of the pairwise Euclidean distances were recorded. The random shuffling was kept on iterating until the convergence (difference <0.01 for >10 iterations). The dendrogram was cut using the converged FDR cutoff. Third, each sub-dendrogram was recursively split until 5th percentile of the pairwise correlation coefficients of genes within the subGO >95th percentile of the randomized background. The randomized background was calculated using Spearman’s correlation matrix of the shuffled relative gene expression levels of the same genes. Finally, we retained the gene modules containing ≥20 genes or 5% of genes of the mother GO.

We examined the overlap between subGOs and annotated GO child terms. The subGOs of each GO term were compared to their respective GO-annotated child terms using the Jaccard index


(2) \begin{equation*} \left(\frac{\mid A\cap B\mid }{\mid A\cup B\mid}\right) \end{equation*}

and Sørensen–Dice coefficient


(3) \begin{equation*} \left(\frac{2\mid A\cap B\mid }{\left|A\right|+\mid B\mid}\right), \end{equation*}

where *A* represents the genes in a subGO and *B* represents the genes in a GO child term.

### The comparison with published software to identify coexpression modules

To benchmark our algorithm performance, we implemented two popular coexpression module detection algorithms: Fuzzy clustering by Local Approximation of Membership (FLAME) [17] and Weighted Gene Co-expression Network Analysis (WGCNA) [18]. We included the 28 GO terms we selected in the analyses. For WGCNA, we calculated the Spearman correlation coefficients of pairwise genes in a GO term across cancer cell lines, performed hierarchical clustering of the coefficient values, and calculated the Euclidean distances. The Euclidean distances dendrogram was input to the cutreeDynamic() function with its default parameters. “Unassigned” genes with “0” annotation from the WGCNA output were excluded from further analyses. The gene expression data analysis suite (GEDAS) software (https://sourceforge.net/projects/gedas/) was used to conduct the FLAME analyses. For each GO term, the mean-centered expression matrix was input into the program. “Outlier” genes were excluded from further analyses. For each identified gene module, the median value of Spearman’s correlation coefficients of gene pairs with the module was calculated. The Wilcoxon rank-sum test was performed to calculate the *P*-values comparing our subGO modules versus those obtained from FLAME and WGCNA.

### Calculation of subGO expression index

Given one subGO module with *m* genes, its expression index value in cell line *j* is calculated as


(4) \begin{align*} {I}_j=\text{Median}\left({E}_{1j},{E}_{2j},\dots{E}_{mj}\right)+&\ \text{Median}\left(\left({M}_1-{B}_1\right),\left({M}_2-{B}_2\right)\right.,\nonumber\\&\left.\ldots \left({M}_m-{B}_m\right)\right), \end{align*}

where *E_ij_* is the median-centered expression level (log2) for gene *i* in cell line *j*, *M_i_* and *B_i_* are the median and 5th percentile expression levels of gene *i* across all cell lines, respectively, and *n* is the total number of cell lines included in the analyses. The 5th percentile expression was used as the baseline expression of a gene across cancer cells. Adding $\text{Median}\left(\left({M}_1-{B}_1\right),\left({M}_2-{B}_2\right),\ldots \left({M}_m-{B}_m\right)\right)$ allowed the index values to be mostly positive numbers across cells. A similar approach was also used to calculate the expression index using RNA sequencing data with ${E}_{ij}={R}_{ij}-\text{Median}\left({R}_{i1},{R}_{i2},\ldots{R}_{in}\right)$, where *R_ij_* is the log2(transcript per million (TPM) + 1) value for gene *i* in cell line *j*.

To confirm the coexpression of genes within a subGO module, we analyzed the human cancer cells’ transcriptomic profiles (measured by microarray) from the Genomics of Drug Sensitivity in Cancer (GDSC) (http://www.cancerrxgene.org/) [19]. For 616 cancer cell lines examined by both CCLE and GDSC, we calculated the subGO expression index and examined their correlation using the two independent datasets.

### Examining the correlation between subGO expression and cancer cell’s metastatic potentials

The cancer cells’ metastasis potential data were obtained from the MetMap project (https://depmap.org/metmap/) [8]. For each metastasis route from one lineage origin to a destination, we calculated Spearman’s correlation coefficients between the metastatic potentials and a subGO expression index across cancer cells. Then, we performed the hierarchical clustering of the coefficient values to examine the similarity between different metastatic routes and subGO modules. Seven coherent subGO clusters were identified, and each cluster showed a recurrent positive correlation with metastatic potentials across multiple routes. We focused on metastatic routes showing a positive correlation with at least one subGO cluster for further analysis.

For a cancer lineage type, the cells’ metastatic potentials toward different destinations are positively correlated, except for pancreatic cancers. For these lineage types, we merged different metastatic destinations for the downstream analyses. For pancreatic cancers, we analyzed their metastasis to brain/bone and lung, separately. The averaged correlation for a lineage type was calculated as previously described [20]. The correlation coefficient $r$ was coverted into a Fisher’s $z$:


(5) \begin{equation*} {z}^{\prime }=0.5\ \ln \frac{1+r}{1-r}\ . \end{equation*}

Then, we took averaged $z$ score:


(6) \begin{equation*} \overline{z}=\frac{\sum{Z}^{\prime }}{n}. \end{equation*}

And finally the $\overline{z}$ score was converted back to $r$:


(7) \begin{equation*} \overline{r}=\frac{e^{2\overline{z}}-1}{e^{2\overline{z}}+1}. \end{equation*}

We used a one-tailed *t*-test to examine whether the complied Spearman’s correlation coefficient values for a subGO cluster were >0 for a lineage type. A cluster was considered to be significantly associated with metastatic potentials of cancer cells from a lineage type if the *t*-test *P*-value <.01 and the averaged coefficient value >0.15.

### Random forest regression modeling using the subGO expression levels to predict metastatic potentials

We built a regularized random forest model for each metastasis route. We used the RandomForestRegressor() in the sklearn.ensemble class of Scikit-learn Python package (version 0.24.2) to build the random forest regression model. Three-fold cross-validation was applied to identify the optimal tree numbers (from 1 to 500, in intervals of 5), max tree depth (from 1 to 50, in intervals of 1), and the number of features to identify the best split. Tree numbers, max tree depth, and node splitting feature numbers were set using “n_estimators”, “max_depth”, and “max_features” parameters, respectively. All parameter combinations were grid searched, and the model with the lowest mean squared error between the observed and predicted values was then applied to train a model with all data points. Grid search for the hyperparameter tuning was implemented using the GridSearchCV() in the sklearn.model_selection class of Scikit-learn Python package (version 0.24.2).

The leave-one-out validation [21] was employed to assess the robustness of the models. For each random forest model, samples were iteratively excluded one cell line at a time from all the training data. This process resulted in the creation of *n* − 1 leave-one-out validation models, where *n* represents the total number of cell lines. Pairwise Spearman’s correlation coefficients of the Gini index were calculated to examine the model generalization. Correlations of the Gini index between leave-one-out models indicated the robustness of our models.

To evaluate the performance of our random forest regression model predictions, we examined the correlation between the observed metastatic potentials across routes versus the model-predicted values. We used the receiver operating characteristic (ROC) curve to measure the performance of using predicted values to classify metastatic (observed value > −2) versus non-metastatic (observed value ≤ −2) cancer cells.

### The analyses of protein–protein interaction network

To identify the hub genes in each subGO cluster, we used the STRING v.12.0 database (https://string-db.org/) [22] to examine the protein–protein interaction and coexpression network. We focused on the three high-confidence interactions, including (1) protein–protein interactions detected by biochemical or biophysical assays (Experiments), (2) curated databases annotated protein interaction groups (Databases), and (3) coexpressed genes validated interactions from human sources and those transferred by homology from other species (Co-expression). The Markov cluster algorithm (MCL) was applied to identify the interacted subclusters of genes from each subGO cluster. The biological process with the highest enrichment in each subcluster was used to indicate the subcluster name. The “inflation” parameter of MCL was fine tuned to ensure each subcluster was enriched with genes from different biological pathways. The top enriched subclusters were identified and their associated genes were considered as hub genes for each subGO cluster.

For the visualization, we showed the edges between genes supported by STRING-defined interactions. The edge color was defined by Spearman’s correlation coefficient value between two genes across CCLE cancer cell lines.

### Analyses of clinical and transcriptomic data of TCGA

The RNA-Seq gene expression data were downloaded from the GDC Data Portal (https://gdc.cancer.gov). The patient survival data were obtained from the TCGA Pan-Cancer Clinical Data Resource [23]. For each subGO cluster, the index was calculated using the expression data of interacted hub genes. Then the patient was divided into two groups based on the module index (top 50% versus low 50%). Cox regression was conducted to obtain the direction of prognosis and the log-rank test was used to determine the significance *P*-value of patients’ overall survival rates. The analyses were conducted using the R package “survminer” (version 0.4.9).

### Analyses of scRNA-seq data

The scRNA-seq data for melanoma and liver cancer patients were obtained from GSE115978 [24] and CNP0000650 [25], respectively. The expression for gene *i* in cell *j* was calculated as


(8) \begin{equation*} {Er}_{ij}=\log 2\left({CPM}_{ij}/10+1\right), \end{equation*}

where the count per million (*CPM_ij_*) value was calculated as


(9) \begin{equation*} {CPM}_{ij}=\frac{10^6\times{UMI}_{ij}}{\sum_{k=1}^m{UMI}_{kj}} \end{equation*}

for gene *i* in cell *j*, with *m* being the total gene number. Due to the dropout issue, scRNA-seq can only capture ~10% of the whole transcriptome. Dividing the $CPM$ values by the scale factor 10 was applied to ensure appropriate normalization and avoid inflating the differences between detectable and undetectable genes [26, 27]. Genes with ${Er}_{ij}$ > 3.5 in at least 5% of cells were included. ${Er}_{ij}$ was then mean centered for further analyses:


(10) \begin{equation*} {E}_{ij}={Er}_{ij}-\text{Mean}\left({Er}_{i1},{Er}_{i2},\ldots{Er}_{in}\right). \end{equation*}

Cells with <1000 unique molecular identifiers were excluded.

The module index calculation for scRNA-seq was slightly different from the method we used for bulk-RNA expression. Due to the sparsity of scRNA-seq data and some genes showing the expression level of “0” in a cell due to the dropouts, we used the mean instead of median expression to normalize the data across single cells. Given one gene module with *m* genes, its index value for the cell *j* is calculated as


(11) \begin{align*} {I}_j=\text{Mean}\left({E}_{1j},{E}_{2j},\ldots{E}_{mj}\right)+&\ \text{Median}\left(\left({M}_1-{B}_1\right),\left({M}_2-{B}_2\right),\right.\nonumber\\&\left.\ldots \left({M}_m-{B}_m\right)\right) \end{align*}

where *E_ij_* is the mean-centered expression level for gene *i* in single cell *j*, while *M_i_* and *B_i_* are the median and 5th percentile expression levels of gene *i* across all single cells, respectively.

### Analyses of the correlation between drug sensitivity and network expression

The GDSC database provides a collection of IC50 values from 265 molecules, representing the drug concentration required to inhibit cell growth by 50%. To investigate the relationship between drug sensitivity and the index of metastasis-correlated networks, we calculated the correlation coefficient values and the regression *P*-values between log(IC50) values of each drug versus the network index based on hub genes from each subGO cluster across cancer cells.

## Results

### Select biological processes related to cancer metastasis

Dedicated efforts have curated genes regulating different biological processes into Gene Ontology (GO) terms [28, 29]. To identify metastasis-related GO terms, we performed differential gene expression analyses comparing metastatic versus non-metastatic cells from the MetMap project. We examined 17 lineage types with >10 cell lines analyzed by MetMap (Table S1). For each lineage type, we considered a cell type to be metastatic if its metastatic potential is > −2 toward any one of the five destinations (brain, lung, liver, kidney, and bone). The expression levels of individual genes are quite variable across cell lines even for those within a metastatic or non-metastatic group. We calculated the averaged expression differences between metastatic versus non-metastatic cells. To identify molecular pathways promoting cancer metastasis, we performed the GO analyses of the genes showing higher expression in metastatic cells (top 1000 genes) for each lineage type using the DAVID software [30]. The cutoffs to select differentially expressed genes are shown in Table S1, and the GO analysis results are in Table S2.

The cancer-promoting pathways were summarized into the 10 hallmarks of cancer, and the top enriched biological processes from our GO analyses were highly related. We manually curated a list of 28 GO terms related to each hallmark (Fig. 1B). For example, we used the genes in “GO:0012501 programmed cell death” to represent regulators of the hallmark “resistant cell death”. The term “GO:0001944 vasculature development” was used to identify regulators of “inducing angiogenesis”. The terms such as “GO:0007155 cell adhesion”, “GO:0048870 cell motility”, “GO:0001837 epithelial to mesenchymal transition (EMT)”, and “GO:0000902 cell morphogenesis” were used to examine the regulation of “activating invasion & metastasis”. Additionally, to obtain regulators of oncogenic pathway expression, we included five biological processes mediating steps of the gene expression in the analyses: “GO:0006355 regulation of transcription, DNA-templated”, “GO:0006396 RNA processing”, “GO:0009451 RNA modification”, “GO:0006412 translation”, and “GO:0031399 regulation of protein modification process”.

For each lineage type, we examined the top 20 biological processes enriched with genes showing higher expression in metastatic cells. On average, 87.9% of genes in these pathways (SD = 0.104) belonged to two out of our selected 28 GO terms, and 96.5% (SD = 0.04) were included if considering all GOs. These data indicated that our curated list of GO terms contained the majority of metastasis-related genes or pathways, which we will characterize in detail later.

### An unsupervised clustering approach to identify coexpressed subGO modules regulating biological processes

Although our above analyses identified GO terms related to cancer progression and metastasis, it remains unknown which genes in a GO term are coexpressed across cancer cells. To this end, we developed an unsupervised clustering approach to identifying the coexpressed subGO modules by analyzing the transcriptomic profiles across 1037 human cancer cell lines from >20 lineage types from the CCLE database.

**Figure 2:**
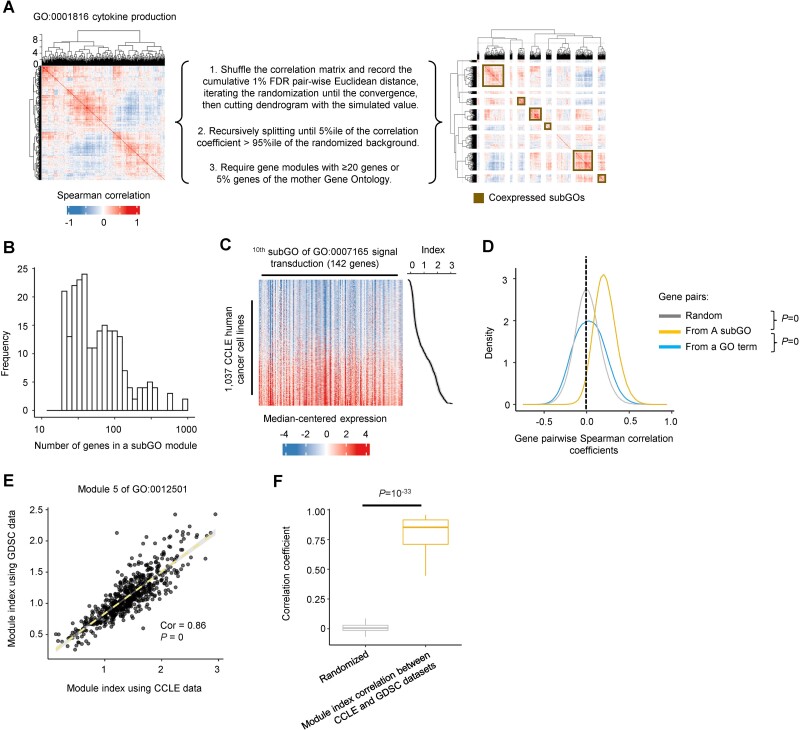
Identifying the coexpressed subGO modules. (A) An example workflow to identify coexpressed subGO modules in the GO term GO:0001816 cytokine production. For the heatmap, each row represents one gene and the Spearman correlation coefficient values between expression levels of gene pairs across CCLE cell lines are shown. (B) Histogram showing the number of genes in 230 identified subGO modules. (C) Heatmap showing the relative expression of 142 genes in a subGO module of GO:0007165 signal transduction. The cancer cells were sorted by the calculated module index values. (D) Histogram showing the pairwise correlation coefficients of gene expression across 1037 human cancer cell lines from 230 subGO modules versus random gene pairs. The Wilcoxon rank-sum test *P*-values are shown comparing the coefficient differences between gene pairs within a subGO module versus random gene pairs or those from a GO term. (E) Scatter plot showing the expression of a subGO module of GO:0007165 signal transduction calculated using CCLE and GDSC datasets. Each dot represents a cell line and there are a total of 616 intersected cell lines. The Pearson correlation coefficient and regression *P*-value are shown. (F) Boxplot showing the correlation coefficients between GDSC and CCLE index of the 230 subGO modules. The randomized group shows expression correlation coefficients between randomly paired gene modules. The Wilcoxon rank-sum test is used to examine the statistical differences.

We used the following analysis steps to obtain the coexpressed subGO modules. For genes in a GO term, we performed hierarchical clustering of Spearman’s correlation coefficients of pairwise gene expression levels across the cancer cells. Then, the hierarchical clustering dendrogram was cut with the simulated 1% FDR cutoff from the randomly shuffled expression data (Fig. S1A; see Methods for details). We further required the 5th percentile of Spearman correlation coefficient values of genes in a subGO to be >95th percentile of those calculated using the shuffled data by further cutting down the dendrogram. Finally, we required the number of genes in the retained subGOs should be >20 or 5% of the total genes in the GO term. We showed the analysis steps for an example GO term “GO:0001816 cytokine production” in Fig. 2A.

Using the approach, we identified 230 coexpressed subGO modules across the 28 selected GO terms (Table S3). The subGO modules were with varying sizes of 20 to 884 genes, with a median gene number of 49 (Fig. 2B). The expression correlation coefficients of gene pairs within modules were significantly higher than random gene pairs (Wilcoxon rank-sum test *P*-value = 0) (Fig. 2CD). We also compared our algorithm performance with published software. The genes within subGOs we defined show significantly higher expression correlation than those from the modules identified by FLAME [17] or WGCNA [18] indicating the better performance of our algorithm (Fig. S2).

The coexpression pattern of a gene module was consistent across individual lineage types (Fig. S1B). Different numbers of subGO modules were obtained from GO terms, reflecting the diverse complexities of associated genes (Fig. S1C). For example, we obtained 12 subGOs from “GO:0009605 response to external stimulus” and 4 subGOs from “GO:0006091 generation of precursor metabolites and energy” (Fig. S1D). Most subGOs contained modest overlapping genes with annotated GO child terms shown by the Jaccard index and Dice–Sørensen coefficients (Fig. S1EF and Table S4). The results indicated that our subGO modules do not simply represent specific GO offspring.

In a cancer cell, we calculated the expression index of a subGO module as the median relative expression across genes in the module (Fig. 2C, see Methods for details). To examine the robustness of our module index calculation across cancer cells, we analyzed an independent gene expression dataset from the GDSC database. For the 616 cell lines characterized by both databases, the module index values inferred in CCLE were significantly correlated with those from the GDSC (Figure 2EF, median correlation coefficient value = 0.86 and *P* = 10^−33^ compared to the random genesets), indicating the robustness of our module index calculation across cancer cells.

### The subGOs form seven clusters which are correlated with cancer cells’ metastatic potentials across different lineage types

**Figure 3:**
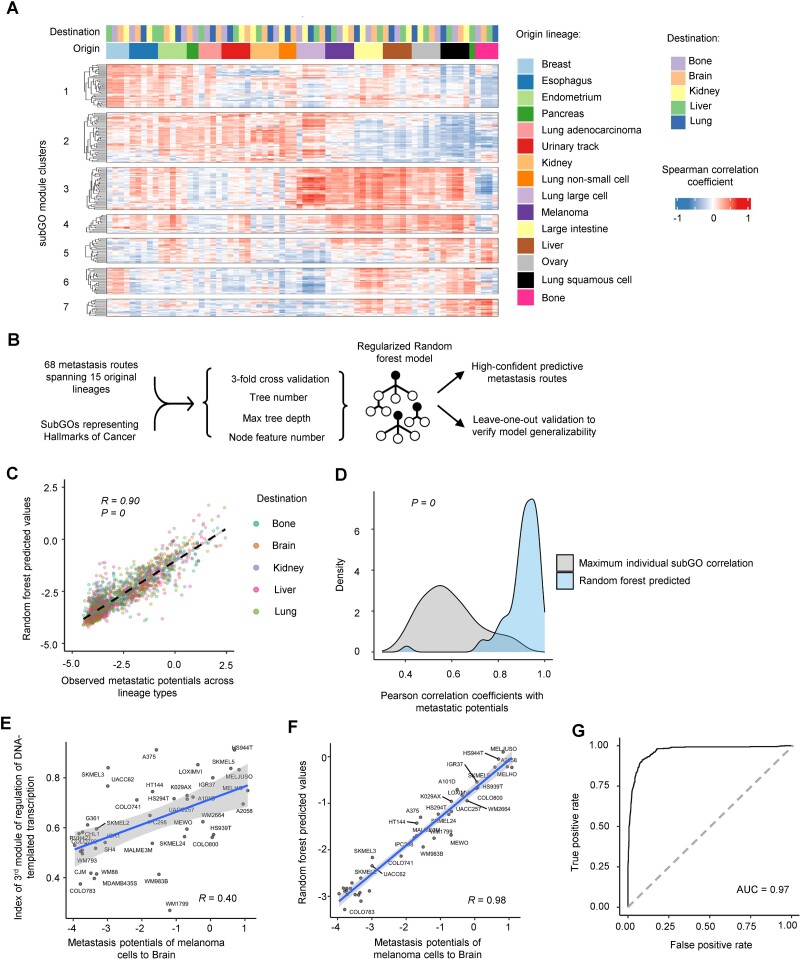
The intrinsic expression levels of subGO modules in cancer cells are correlated with their metastatic potentials *in vivo*. (A) Heatmap showing Spearman’s correlation coefficient values between subGO index and metastasis potentials. Each column shows a metastatic route from the indicated origin to the destination. We performed the hierarchical clustering and found that the subGO modules form 7 clusters as indicated. (B) The workflow to build a regularized random forest model to use the subGO module expression to predict the metastatic potentials across different routes. (C) Scatter plot showing the Pearson correlation between observed metastasis potential versus random forest predicted values across 69 metastasis routes. Each data point on the plot represents a specific metastasis route. (D) Density plot showing the distribution of correlation coefficient values between metastatic potentials versus individual subGO expression and the random forest predicted values. (E) Scatter plot showing the correlation between malignant melanoma to brain metastatic potentials and the expression of a module of “regulation of DNA-templated transcription”. Each dot represents one cell line. (F) Scatter plot showed the correlation between malignant melanoma to brain metastatic potentials and the random forest predicted values. Each dot represents one cell line. (G) The ROC curve shows the performance using the random forest predicted values to classify the metastatic versus non-metastatic cancer cells. The area under the ROC curve is shown.

Next, for each lineage, we calculated the Spearman correlation coefficient between subGO module expression and metastatic potentials across cancer cells. We performed the hierarchical clustering of the metastatic routes and subGOs based on the coefficient values (Fig. 3A and Table S5). The subGOs formed seven clusters, and each cluster showed a recurrent positive correlation with metastatic potentials across multiple lineage types (Fig. 3A). Within each cluster, the subGOs regulating different biological pathways generally showed a positive correlation of expression (Fig. S3A). For 90 metastatic routes we examined, 68 (86.3%) from 15 lineage types showed a positive correlation with at least one subGO cluster (Fig. 3A). We did not observe a significant correlation for the remaining 22 routes (Fig. S3B).

We further examined whether the cancer cells’ intrinsic pathway expression represented by subGO module index values can predict their metastatic potentials *in vivo*. To this end, we developed a regularized random forest regression model using the subGO module index values as the input to predict the cancer cells’ metastatic potential for each route (Fig. 3B). To avoid overfitting, 3-fold cross-validation was applied to regularize and optimize the hyperparameters of each model (see Methods for detail). We used the leave-one-out validation approach to examine the robustness of our regularized model. The Gini indices from the different models (randomly leaving one sample out) were well correlated, indicating the models’ robustness (Fig. S4A). In contrast, if we did not apply regularization, the Gini indices values were not correlated from the different models suggesting the overfitting (Fig. S4A).

We built a random forest regression model for each of the 68 metastatic routes from 15 lineage types shown in Fig. 3A. Our models integrated the subGO expression features and generated a predictive value for each route (Table S6). Considering all the routes, the overall Pearson correlation coefficient between the predicted and observed values was 0.90 (Fig. 3C; *P*-value = 0). The random forest model-predicted values showed a higher correlation with metastatic potentials than individual subGO expression indexes (Fig. 3D–F and Fig. S4BC). Based on the cutoff −2 defined by the previous study [8], our predicted values effectively classified metastatic versus non-metastatic cells with an area under the receiver operating characteristic value of 0.97 (Fig. 3G). Altogether, by developing the random forest regression model, we showed that the expression of subGO modules we defined in cancer cells can effectively predict their metastatic potentials *in vivo*.

**Figure 4:**
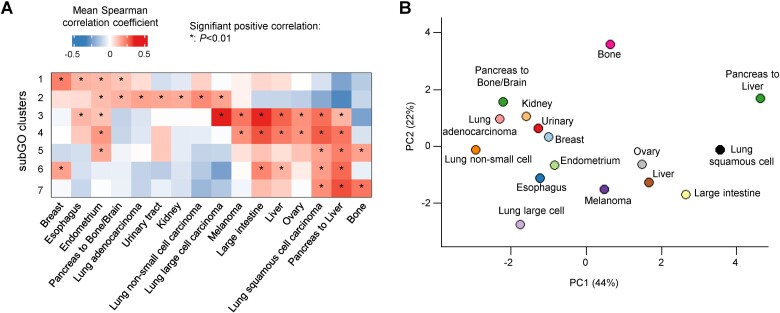
Identifying the gene module clusters mediating metastatic potentials across lineage types. (A) For most cancer lineage types (except pancreatic), their metastatic potentials toward different destinations are positively correlated. For each module cluster, we calculated the averaged Spearman correlation coefficient values across destinations and used the *t*-test to determine whether the complied coefficient values were significantly >0. For pancreatic cancers, we split them into two groups, including metastasis to bone/brain and to liver, respectively. Heatmap showing the averaged Spearman’s correlation coefficient values. We considered a cluster to be significantly positively correlated with a metastatic route if the *P*-value <.01 and the coefficient value >.15. (B) The principal component analyses of metastatic potential-correlated module clusters across lineage types using the input values from (A). The percentages of total variance explained by the principal components are shown in parentheses.

For most lineage types except pancreatic cancers, the cancer cells’ metastatic potentials toward different organs (i.e. brain, bone, liver, kidney, and lung) were positively correlated (Fig. S5). Also, the correlated subGOs were consistent across different metastatic destinations (Fig. 3A). For these lineages, we merged the correlation values to different organs and examined whether the correlation coefficient values were significantly >0 using the *t*-test. A subGO cluster was considered to be significantly correlated with metastatic potentials if the *t*-test *P*-value was <0.01 and the mean coefficient value was >0.15 (Fig. 4A). For pancreatic cancers, the cells’ metastatic potentials to the liver exhibited a different pattern versus those toward bone and brain (Fig. S5), and we separated into two groups during the later analyses. Altogether, our analysis identified the subGO clusters linked to different molecular pathways showing recurrent positive correlation with cancer cells’ metastatic potentials across lineage types (Fig. 4AB).

### Gene modules predicting cancer cells’ metastatic potentials are conserved *in vivo* and are correlated with cancer aggressiveness

**Figure 5:**
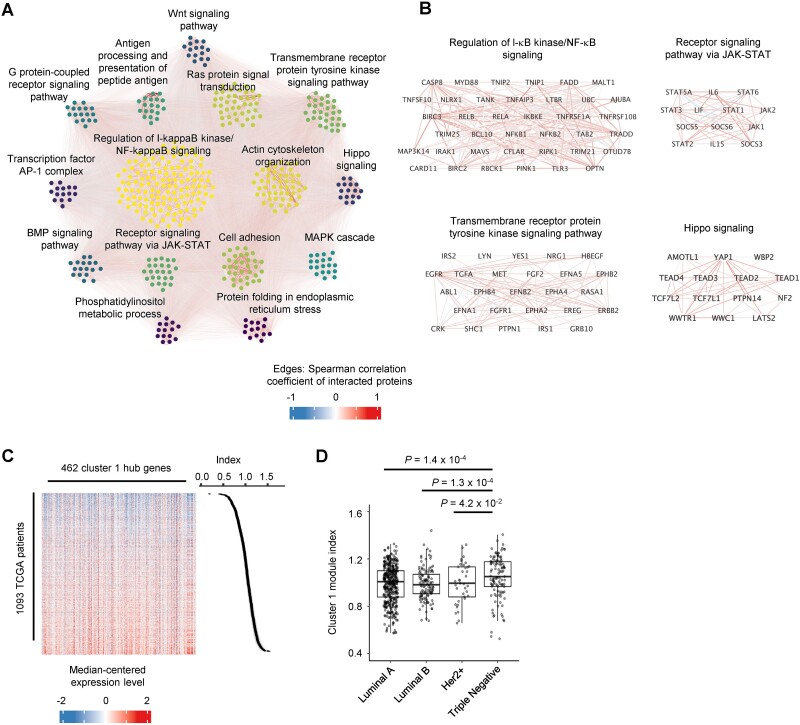
The analyses of the gene regulatory network of module cluster 1. (A) The interaction network of module cluster 1. The gene subclusters were defined by the MCL analyses of the STRING database, and the subcluster name represents the most enriched biological process. The edges represent the interaction nodes and the gradient is measured as the Spearman correlation coefficient of gene expression across cancer cell lines from CCLE. (B) The interaction network showing example genes in indicated subclusters. (C) Heatmap showing the relative expression of hub genes in (A) across breast cancer samples from TCGA. We calculated the module gene expression as the index value for each patient. (D) The relative expression of cluster 1 gene module in breast cancer patients grouped by their genetic subtypes: triple negative (ER−, progesterone receptor − (PR−), and HER2−), luminal A (ER+, PR+, and HER2−), luminal B (ER+, PR+, and HER2+), and HER2+ (ER−, PR−, and HER2+).

The expression of cluster 1 subGOs was correlated with the metastatic potentials of breast, esophagus, endometrium cancers, and pancreatic cancers to bone/brain (Fig. 3A and Fig. 4A). To obtain the regulatory modules in the cluster, we used the STRING database [22] to examine their protein–protein interactions and employed the MCL algorithm to identify enriched gene subclusters (see Methods for details). We identified 15 subclusters with ≥14 genes (Fig. 5A), and each subcluster was associated with genes linked to different oncogenic pathways (Fig. S6A, Table S7, and the network files are deposited at https://github.com/zhejilab/MetNet). The network includes well-characterized oncogenic pathways, such as the I-κB kinase/NF-κB signaling (e.g. TNFRSF1A, IKBKE, RELA, RELB, NFKB1, and NFKB2), the JAK–STAT3 pathway (e.g. IL6, JAK1, JAK2, STAT3, and STAT6), the Hippo pathway (e.g. YAP1, TEAD1, TEAD3, and TCF7L2), cell adhesion genes (e.g. CD44, LAMB1, ITGA3, and COL4A5), the tyrosine kinase signaling (e.g. ABL1, ERBB2, FGF2, and MET), and AP-1 transcription factors (e.g. JUN, JUNB, and FOS) (Fig. 5AB and Fig. S6B). Intriguingly, some peptide antigen processing and presentation genes were also part of the coexpressed network, such as HLA-A, HLA-F, HLA-B, and HLA-C (Fig. S6AB). A coexpressed subGO module was the classical metastasis-promoting pathway “epithelial to mesenchymal transition” with genes such as TWIST1, SNAI2, TGFB1, and HIF1A (Table S5).

We analyzed the RNA-seq data from TCGA [31] and calculated the network expression levels based on the hub genes defined above across 1093 breast cancer patients (Fig. 5C). The cluster 1 gene network defined above has higher expression in triple-negative breast cancer compared to estrogen receptor-positive (ER+) and HER2+ subtypes indicating the relevance in patients (Fig. 5D). Many of these genes such as those in the NF-κb, STAT3, and YAP pathways, have been shown to drive breast cell transformation and cancer metastasis [32–34]. Our analyses effectively identified these known regulators. Here, we showed that the network may mediate the metastatic potentials of multiple cancer types, including endometrium, esophagus, and pancreatic cancers (Fig. 4A).

The metastatic potentials of lung adenocarcinoma, kidney, urinary tract, and pancreatic cancers were correlated with the expression of the gene cluster 2. The analyses of the protein–protein interaction network showed that the genes were enriched in the pathways, such as small GTPase-mediated signal transduction (e.g. VAV1, RAC1, and NCKAP1), protein tyrosine kinase signaling pathway (e.g. EGFR, YES1, PTK2, and EFNA1), regulation of cytokine production (e.g. NOD1, RIPK2, and ITCH), regulation of response to stimulus (e.g. TANK, TRAF2, and IRAK4), and unsaturated fatty acid metabolic process (e.g. CYP1A1, GSTP1, and CYP2A6) (Fig. S7, Table S7, and the network files are deposited at https://github.com/zhejilab/MetNet). The analyses of TCGA data showed that lung adenocarcinoma patients with higher expression of cluster 2 network show worse survival rates. For pancreatic cancers, the higher expression of both cluster 1 and cluster 2 networks are also correlated with worse patient survival (Fig. S8), indicating the clinical relevance of the gene networks.

**Figure 6:**
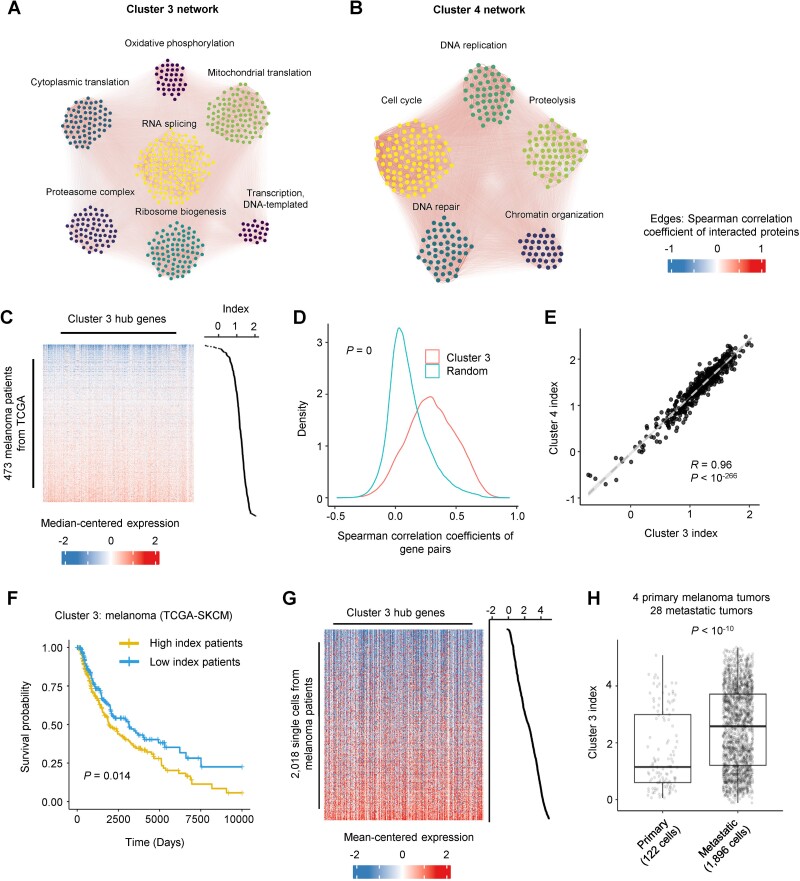
The gene regulatory network of module clusters 3 and 4. (A) The interaction network of module cluster 3. (B) The interaction network of module cluster 4. (C) Heatmap showing the relative expression of hub genes in cluster 3 across melanoma patients from TCGA. We calculated the module gene expression as the index value for each patient. (D) The Spearman correlation coefficients of gene pairs from cluster 3 and random pairs. The Wilcoxon rank-sum test *P*-value is shown. (E) The correlation between cluster 3 and cluster 4 expression across melanoma patients. And the regression *P*-value is shown. (F) The Kaplan–Meier estimator shows the melanoma patients with high (top 50%) versus low (low 50%) cluster 3 network expression. The log-rank test *P*-value is shown. (G) Heatmap showing the relative expression of cluster 3 hub genes across 2018 single cells from melanoma cells. We calculated the module gene expression as the index value for each cell. (H) Boxplot comparing the expression of cluster 3 gene module across single cells from primary and metastatic melanoma tumors. The Wilcoxon rank-sum test *P*-value is shown.

The metastatic potentials of melanoma, liver, ovary, large intestine, and lung squamous cell carcinoma were correlated with the expression of clusters 3 and 4 (Fig. 3A and Fig. 4A). We identified the protein–protein interaction network in the two clusters using the similar approach described above. The cluster 3 genes were enriched in the following pathways such as RNA translation (both mitochondrial and cytosolic translation factors), RNA processing (e.g. HNRNPM, U2AF2, and SF3B1), ribosome biogenesis (e.g. WRD43, NOP56, and NOL6), protein ubiquitination (e.g. PSMB6, PSMD3, and PSMB2), oxidative phosphorylation (NDUFA6, COX6B1, and NDUFAB1), and general transcription factors (e.g. POLR2L, GTF2H1, and POLR2G) (Fig. 6A, Fig. S9, Table S7, and the network files are deposited at https://github.com/zhejilab/MetNet). The cluster 4 genes were enriched in pathways of the cell cycle (e.g. CDC6, CCNB2, and CDK2), DNA replication (e.g. MCM10, ORC6, and CHTF18), DNA repair (e.g. MSH2, FANCD2, and MSH6), proteolysis (UBE2N, USP14, and NEDD8), and chromatin organization (e.g. SMARCA5, NASP, and BAP1) (Fig. 6B, Fig. S10, and the network files are deposited at https://github.com/zhejilab/MetNet). For cluster 3 and cluster 4, their correlation with metastatic potentials was generally consistent across different lineage types, except for the lung large cell carcinoma (Fig. 3A and Fig. 4A).

We further analyzed the transcriptomic profiles of cancer patients from TCGA to examine the expression of clusters 3 and 4 *in vivo*. Using the RNA-seq data for 473 melanoma, 470 liver cancer, and 737 colon cancer patients, the genes from clusters 3 and 4 were coexpressed *in vivo* (Fig. 6C–E, Fig. S11A–C, Fig. S12A–C, Fig. S13A). The patients with higher expression of the gene modules showed worse overall survival rates (Fig. 6F, Fig. S11C–E, and Fig. S12DE, Fig. S13B). Moreover, we analyzed the single-cell RNA sequencing data from the metastatic and primary melanoma and liver tumors [24, 25]. The metastatic cells showed higher expression of clusters 3 and 4 genes compared to non-metastatic cells (*P* < 10^−10^ comparing 1896 metastatic versus 122 primary melanoma cells, and *P* < 10^−37^ comparing 304 metastatic versus 2433 primary liver cancer cells) (Fig. 6GH, and Fig. S11FG, Fig. S12F–I). The data indicated the conservation of gene networks from clusters 3 and 4 in cancer patients. The gene networks from clusters 5, 6, and 7 are described in Figs. S14, S15, and S16, respectively (the network files are deposited at https://github.com/zhejilab/MetNet). Their expression levels were correlated with fewer metastatic routes (Fig. 4A). Altogether, our analyses identified different gene networks that may promote the metastatic potentials of subsets of cancer lineage types.

### Identify drugs for potential metastatic cancer therapies

**Figure 7:**
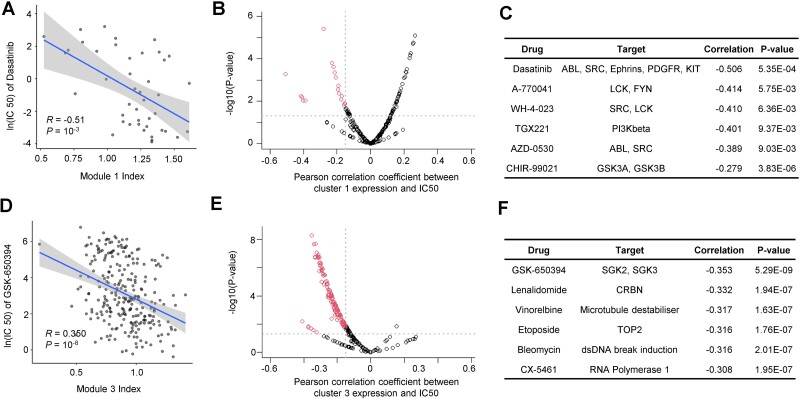
Examine the correlation of drug response and module gene expression. (A) The correlation between dasatinib IC50 versus the cluster 1 network expression levels. (B) Scatter plot showing the Pearson correlation between IC50 and cluster 1 module gene expression versus the −log10(*P*-value) from the regression test. (C) The example drugs whose responses are most correlated with higher expression of cluster 1 gene expression. (D) The correlation between GSK-650394 IC50 versus the cluster 3 network expression. (E) Scatter plot showing the Pearson correlation between IC50 and cluster 3 module gene expression versus the −log10(*P*-value) from the regression test. (F) The example drugs whose responses are most correlated with higher expression of cluster 3 gene expression.

Pre-existing gene signatures within cancer cells play a crucial role in determining their sensitivity to drug treatments [35, 36]. Here, we identified the coexpressed gene modules correlated with metastatic potentials across cancer cell types. Next, we examined whether the drug response measured by the half-maximal inhibitory concentration (IC50) was correlated with the gene network expression (Table S8). When the expression of cluster 1 gene network was higher, the drugs showing significantly lower IC50 values included dasatinib (targeting kinases ABL, SRC, Ephrins, PDGFR, and KIT), A-770041 (targeting LCK and FYN), WH-4-023 (targeting SRC, LCK), TGX221 (targeting PI3Kbeta), and AZD-0530 (targeting ABL, and SRC) (Fig. 7A–C). The cancer cells with high expression of gene module 2 were sensitive to EGFR inhibitors (Fig. S17A–B).

Many drugs showed a negative correlation between IC50 versus cluster 3 and 4 expression which is in line with that they were designed to inhibit cell proliferation (Table S8). The most sensitive drugs included the SGK2 and SGK3 inhibitor GSK-650394, the CRBN inhibitor lenalidomide, the microtubule destabilizer vinorelbine, the TOP2 inhibitor etoposide, the dsDNA break induction drug bleomycin, and the RNA Polymerase 1 inhibitor CX-5461 (Fig. 7D–F). Altogether, our analyses suggested the possibilities for the development of targeted therapies based on the gene signatures we identified in metastatic cancer cells.

## Discussion

Most studies of cancer metastasis have focused on one lineage type for molecular characterization. It remains mostly unknown which lineage types use common or different gene networks. Addressing this question can lead to novel cancer subtyping and targeted therapeutic approaches. In this study, we leveraged a unique MetMap dataset in which they simultaneously measured the metastatic potentials of hundreds of human cancer cell lines using single-cell barcode and sequencing approaches. Through integrated analyses with the transcriptomic profiling data of the cancer cells, we identified and compared gene networks correlated with metastatic potentials across lineage types.

Using an unsupervised approach, we identified coexpressed subGO modules in molecular pathways linked to 10 hallmarks of cancers as well as steps of gene expression. The coexpression of these subGO modules was robust across different lineage types. An advantage of using a gene module index is that it represents a more robust expression of molecular pathways than individual genes. The modules did not simply represent GO-defined child terms and can be a useful resource to examine the gene regulation mediating other biological processes. Using the random forest regression modeling, we showed that the combination of the gene module expression levels in native cancer cells can effectively explain metastatic potentials across different lineage types. Although our analyses were limited by the number of cell lines from a lineage assayed by the MetMap project, the subGOs we identified showed a recurrent positive correlation across multiple metastatic routes from several lineage types. This would not occur by chance and indicated that the gene modules may play regulatory roles in driving cancer metastasis.

We integrated protein–protein interaction with the coexpression to identify hub genes and associated networks in a subGO cluster. The network in cluster 1 modules was enriched with inflammatory regulators, cell adhesion, and pro-survival kinase signaling pathways. The major transcriptional regulators included NF-kb, STAT3, AP-1 factors (e.g. JUN and ATF3), and YAP/TEAD. The gene network is highly similar to that previously characterized in the MCF10A-ER-Src cell transformation model [14, 37–39]. Our data suggested that the inflammatory network promoting breast cell transformation can also enhance the cancer cells’ metastatic potentials of breast, endometrium, esophagus cancers, and pancreatic cancers to bone/brain.

The cluster 4 network was enriched with cell cycle and DNA replication, and the cluster 3 network was with genes regulating gene transcription, RNA splicing, RNA translation, oxidative phosphorylation, and the proteasome pathway. These two clusters showed a general positive correlation of expression across cancer cell lines, presumably because high cell proliferation requires increased rates of gene transcription and protein synthesis. Our data indicated that cell proliferation promotes the metastatic potentials of melanoma, liver, ovary, large intestine, and lung squamous cell carcinoma. We analyzed the transcriptomic profiles from melanoma and liver cancer patients (RNA-seq and scRNA-seq) and showed the conservation and clinical relevance of the networks *in vivo*. Notably, the expression of cluster 1 tends to show a negative correlation with that of clusters 3 and 4 across cancer cells (Fig. S17C). These similarities and differences across lineage types may be determined by their pre-existing gene expression programs.

While our analyses identified gene networks correlated with cancer cells’ metastatic potentials across lineages, a few limitations can be addressed by future experiments. First, the experimental approach taken by the MetMap project was the intracardiac injection of cancer cells into the circulation system which is commonly used in metastasis research. While the measured metastatic potentials represented the capacities of cancer cells to survive circulation, extravasation, and colonization, they did not capture the first two steps of cancer metastasis including invasion and intravasation [7]. Second, the human cell lines and immunodeficient mice were used for the experiments. Future studies using mouse cancer cells in an immunocompetent environment can reveal the roles of the immune system in regulatory cancer cells’ metastatic potentials. Third, our analyses can benefit from more number of cancer cell lines from a lineage type included in the experiments. We only focused on the most robust molecular pathways showing recurrent correlation with metastatic potentials from multiple lineage types in the analyses. Fourth, this study aimed at identifying common pan-cancer regulatory modules and we required genes included in the analyses to be expressed in >50% of cell lines. Thus, lineage-specific regulatory modules can be missed by the analyses. Future work focusing on the analyses of individual lineage types may identify additional cell type–specific regulatory factors. The integrated computational pipeline we established in this study can represent a general approach to dissecting gene networks and be adapted to future new experimental data.

Finally, we analyzed the IC50 values of 265 compounds profiled by the GDSC database. Cancer cells with high expression of gene networks we identified can exhibit increased sensitivity to specific drugs. While additional studies and validation experiments are required, these gene targets and associated sensitive drugs hold promise for the development of targeted therapies that address the challenges posed by metastatic cancer.

## Conclusion

Overall, our study highlights the unbiased analyses encompassing a wide range of genomic data to unravel the commonalities and differences in gene networks underlying cancer metastasis across developmental lineages. The results can be useful for further investigations into targeted therapies and personalized treatment strategies for metastatic cancers.

**Key Points**

- Performed integrated analyses of metastatic potentials and transcriptomic profiles of 493 cancer cells from 21 lineage types. The large-scale analyses identified different gene networks correlated with metastatic potentials across subsets of lineage types.
- The first to perform comparative analyses of gene networks mediating cancer metastatic potentials across lineage types.
- By developing a regularized random forest regression model, we showed that the combination of the module features in the native cancer cells can predict their metastatic potentials.
- By analyzing transcriptomic profile data from cancer patients, we showed that these regulatory networks are conserved *in vivo*, and contribute to cell heterogeneity and cancer aggressiveness.
- We identified drugs whose response is correlated with the intrinsic expression levels of metastasis-related regulatory networks. These results can potentially be useful for designing personalized treatments for metastatic cancers.

## Supplementary Material

supple_infor_bbae357

TableS1_metmap_cell_bbae357

TableS2_diff_exp_GO_bbae357

TableS3_subGOs_bbae357

TableS4_overlap_bbae357

TableS5_cor_met_bbae357

TableS6_random_forest_bbae357

TableS7_network_bbae357

TableS8_drug_bbae357

## Data Availability

The source codes and generated data are available at https://github.com/zhejilab/MetNet.
