# Supplementary material for: Comparative analyses of gene networks mediating cancer metastatic potentials across lineage types
Source: Brief Bioinform. 2024 Jul 22;25(4):bbae357. doi: 10.1093/bib/bbae357 (PMC11262869; doi:10.1093/bib/bbae357)
Supplement: supple_infor_bbae357 [file supple_infor_bbae357.pdf]

Supplementary information for

Comparative analyses of gene networks mediating cancer metastatic potentials across lineage types

Sheng Wang, Emily K. Stroup, Tingyou Wang, Rendong Yang, and Zhe Ji

This file contains the following information:

Supplementary Figure S1 to Figure S17;

Supplementary table legends.

Figure S1

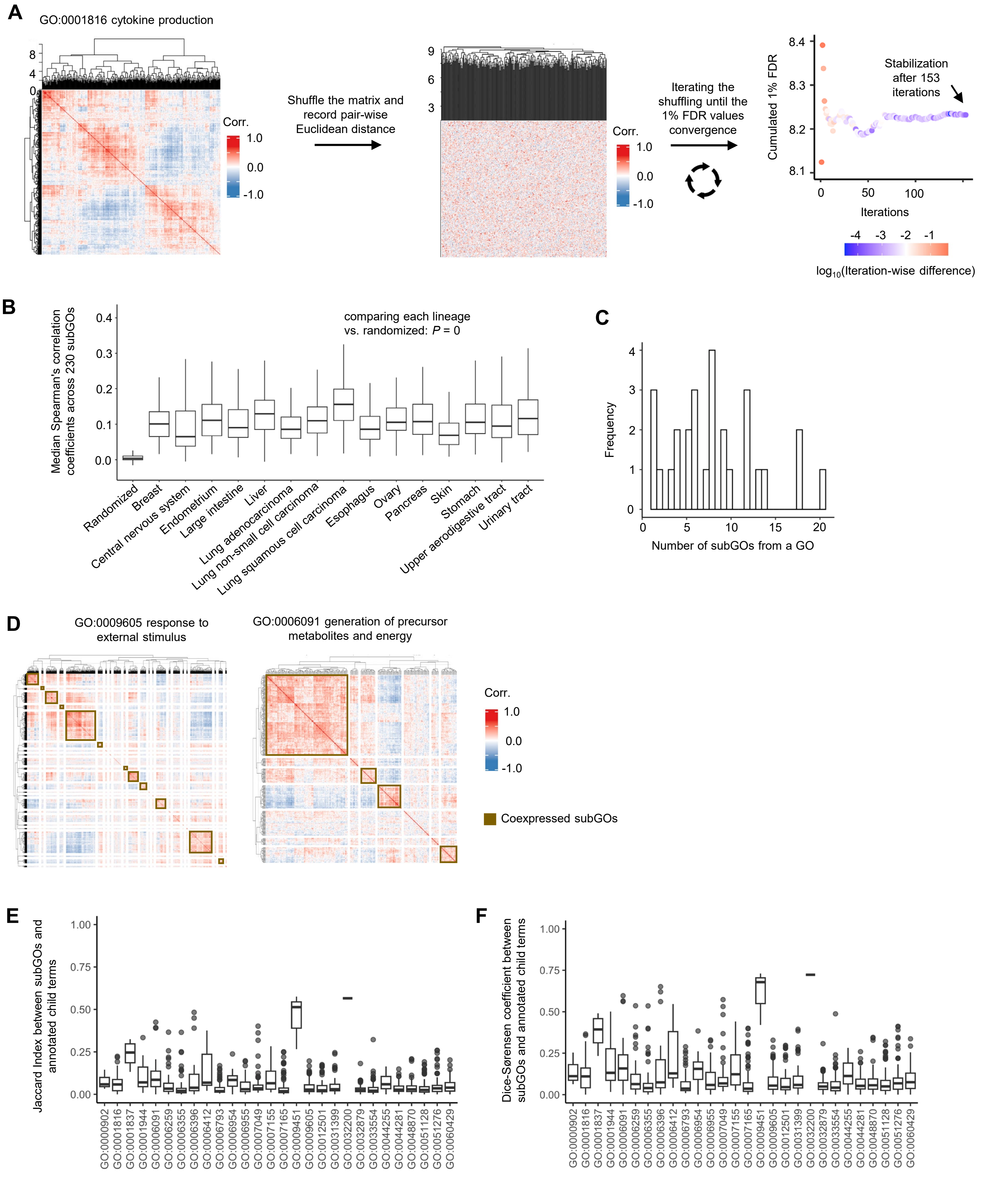

A
GO:0001816 cytokine production
Corr.
1.0
0.0
-1.0
Shuffle the matrix and record pair-wise Euclidean distance
Iterating the shuffling until the 1% FDR values convergence
Cumulated 1% FDR
Stabilization after 153 iterations
Iterations
log10(Iteration-wise difference)
B
Median Spearman's correlation coefficients across 230 subGOs
comparing each lineage vs. randomized: P = 0
Randomized
Breast
Central nervous system
Endometrium
Large intestine
Liver
Lung adenocarcinoma
Lung non-small cell carcinoma
Lung squamous cell carcinoma
Esophagus
Ovary
Pancreas
Skin
Stomach
Upper aerodigestive tract
Urinary tract
C
Frequency
Number of subGOs from a GO
D
GO:0009605 response to external stimulus
GO:0006091 generation of precursor metabolites and energy
Corr.
Coexpressed subGOs
E
Jaccard Index between subGOs and annotated child terms
F
Dice-Sørensen coefficient between subGOs and annotated child terms
GO:0000902
GO:0001816
GO:0001837
GO:0001944
GO:0006091
GO:0006259
GO:0006355
GO:0006396
GO:0006412
GO:0006793
GO:0006954
GO:0006955
GO:0007049
GO:0007155
GO:0007165
GO:0009451
GO:0009605
GO:0012501
GO:0031399
GO:0032200
GO:0032879
GO:0033554
GO:0044255
GO:0044281
GO:0048870
GO:0051128
GO:0051276
GO:0060429

Figure S1. The coexpressed subGOs defined by the study.

(A) Workflow to calculate 1% FDR values of Euclidean distance of randomized gene expression values of a GO term. The Spearman correlation coefficients of gene expression between pairwise genes are shown in the heatmap and each row represents one gene.

(B) The median Spearman's correlation coefficients of gene pairs from 230 defined subGOs across different lineage types. The correlation coefficients of random gene pairs were calculated as the control. The Wilcoxon rank-sum test *P*-value comparing each lineage type vs. the random group is shown.

(C) Histogram showing the number of subGOs defined from a GO term.

(D) The coexpressed subGOs defined in the example GO terms. The heatmaps show the Spearman correlation coefficients of expression levels between gene pairs.

(E) The Jaccard index showing the fraction of overlapped genes from subGOs and annotated GO child terms. The values are shown in Table S4.

(F) The Dice-Sørensen coefficients showing the fraction of overlapped genes from subGOs and annotated GO child terms. The values are shown in Table S4.

Figure S2

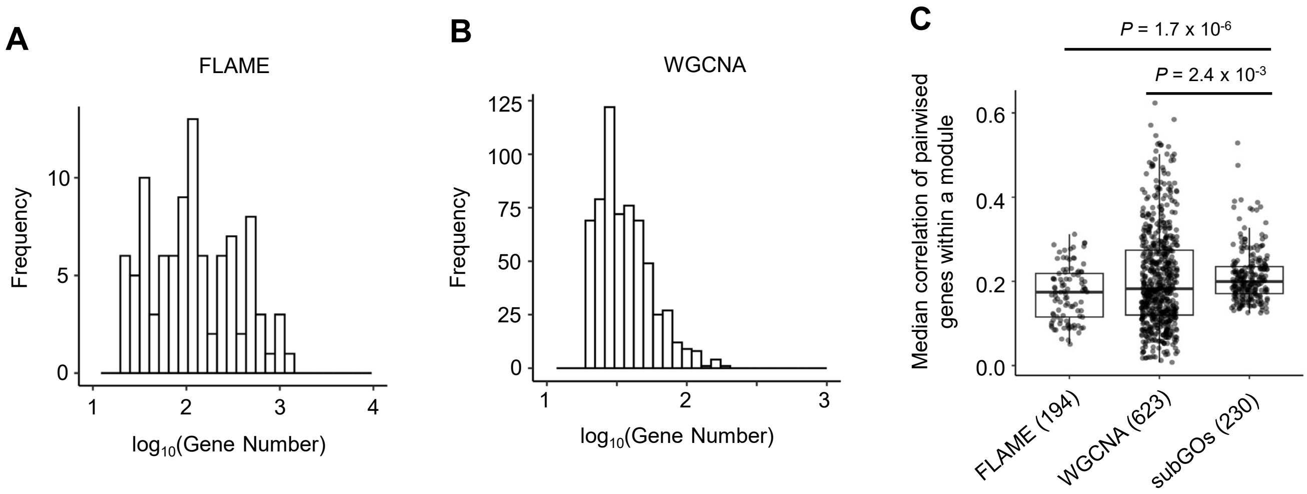

Figure S2. The comparison with other module identification software.
(A) Histogram showing the number of modules from a GO term identified by FLAME.
(B) Histogram showing the number of modules from a GO term identified by WGCNA.
(C) For each module, we calculated the expression correlation values of all gene pairs and used the median value to indicate the overall correlation. Each dot represents one defined module. The Wilcoxon rank-sum test *P*-values comparing values from different groups are shown. The total numbers of gene modules are shown in parentheses.

Figure S3

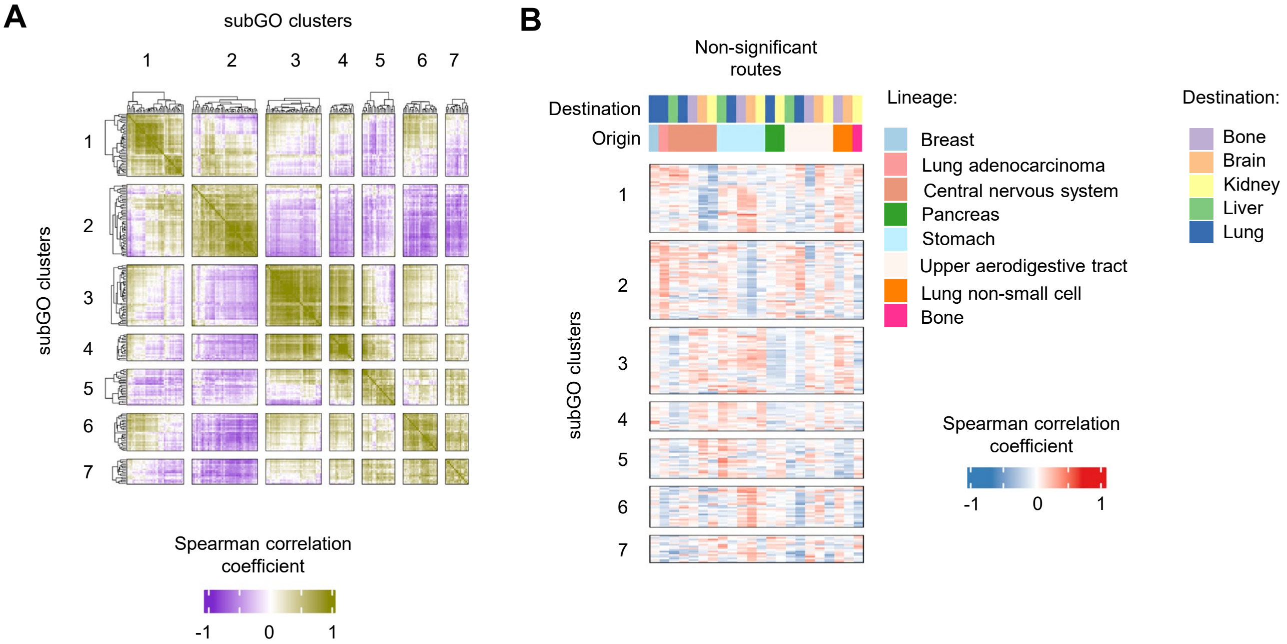

Figure S3. The correlation between subGO expression and metastatic potentials.
(A) The heatmap showing the Spearman correlation coefficients among subGOs from the 7 clusters defined in Figure 3A.
(B) The heatmap showing the correlation between subGO expression and metastatic potentials of indicated routes. The routes that do not show a significant positive correlation with any subGO cluster are shown.

Figure S4

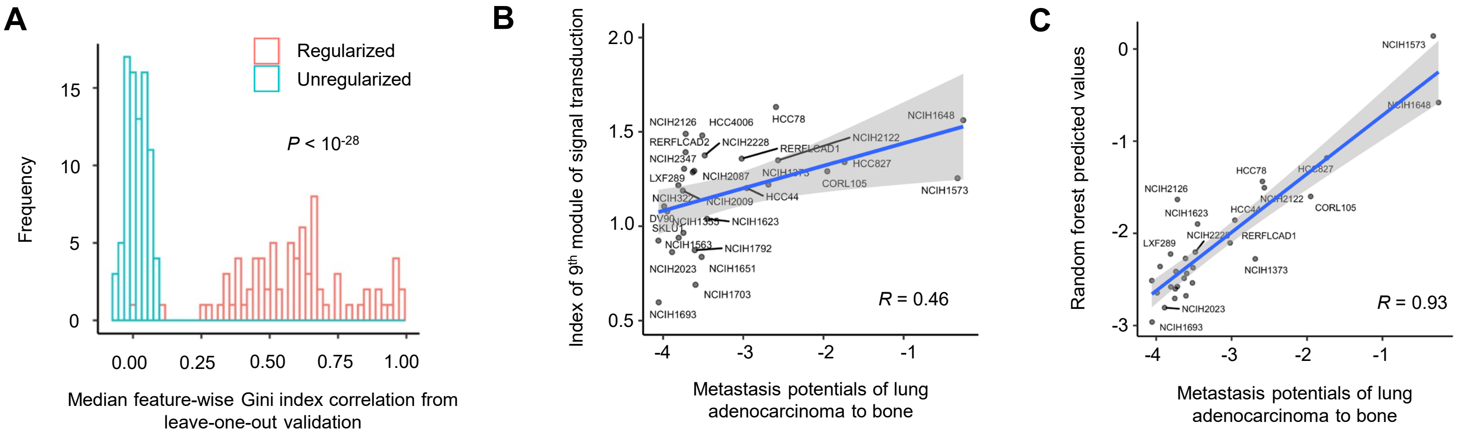

Figure S4. The performance of our random forest model predicting metastatic potentials based on subGO expression.

(A) Leave-one-out validation to verify the generalization of random forest models. Histogram showing the median feature-wise Gini index correlation from leave-one-out validation model trained using regularized and unregularized hyperparameters.

(B) The correlation between the metastasis potentials of lung adenocarcinoma to bone vs. a subGO expression. Each dot represents one cell line.

(C) The correlation between the metastasis potentials of lung adenocarcinoma to bone vs. the random forest model predicted values. Each dot represents one cell line.

Figure S5

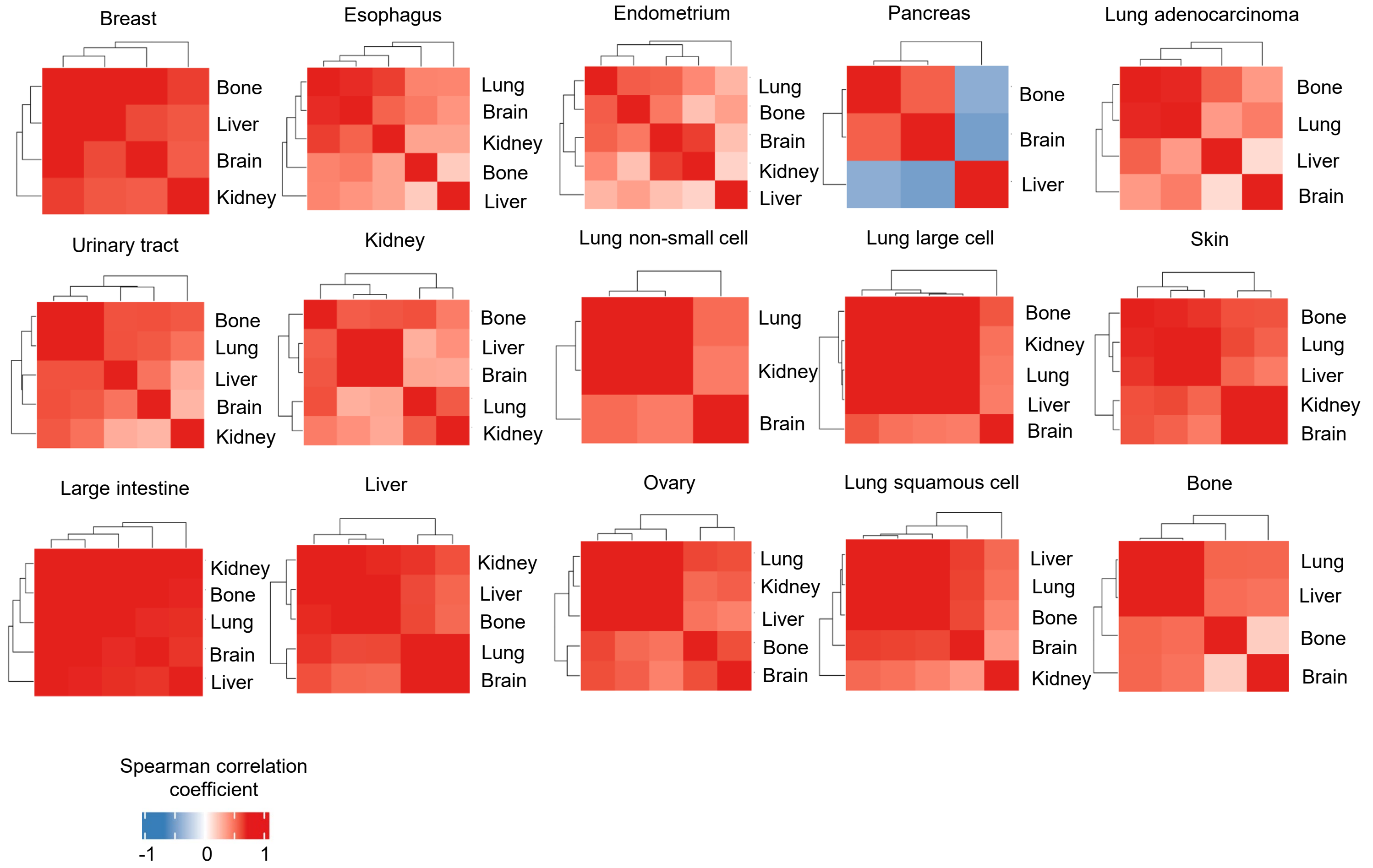

Figure S5. Heatmap showing the correlation of metastatic potentials toward different destinations for each indicated cancer lineage type.

Figure S6

A

Subclusters in cluster 1	GO ID	GO name	Gene number	-log10(p-value)
1	GO:0043122	Regulation of I-kappaB kinase/NF-kappaB signaling	132	32.23
2	GO:0030036	Actin cytoskeleton organization	51	35.32
3	GO:0007265	Ras protein signal transduction	42	14.02
4	GO:0007155	Cell adhesion	34	16.73
5	GO:0007169	Transmembrane receptor protein tyrosine kinase signaling pathway	31	33.60
6	GO:0007259	Receptor signaling pathway via JAK-STAT	25	20.13
7	GO:0048002	Antigen processing and presentation of peptide antigen	19	32.18
8	GO:0000165	MAPK cascade	19	12.33
9	GO:0007186	G protein-coupled receptor signaling pathway	17	14.15
10	GO:0030509	BMP signaling pathway	17	22.63
11	GO:0016055	Wnt signaling pathway	16	22.05
12	GO:0035329	Hippo signaling	16	18.17
13	GO:0046488	Phosphatidylinositol metabolic process	15	13.29
14	GO:0035976	Transcription factor AP-1 complex	14	4.79
15	GO:0034975	Protein folding in endoplasmic reticulum stress	14	14.43

B

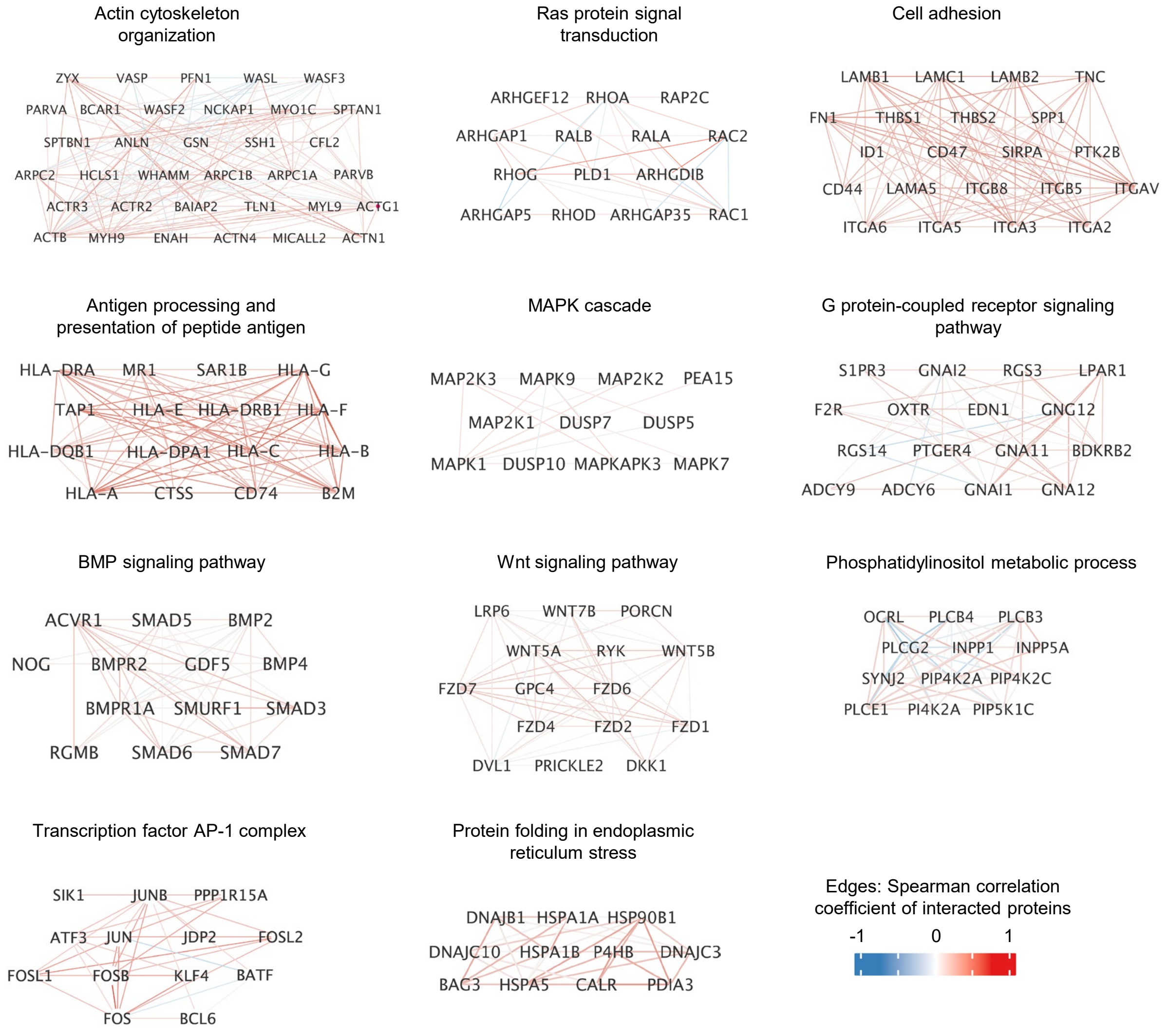

Figure S6. The gene network of subGO cluster 1.
(A) The table showing the most enriched biological process in identified subclusters. The enrichment *P*-values and the total number of genes in each subcluster are shown.
(B) The interaction network showing example genes in indicated subclusters.

Figure S7

A

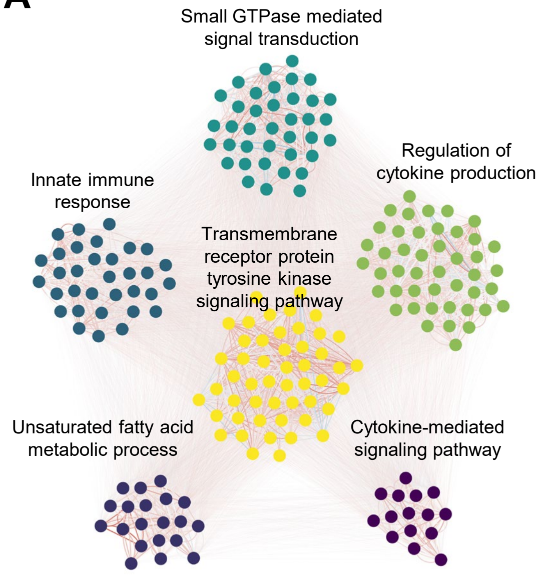

B

Subclusters in cluster 2	GO ID	GO name	Gene number	-log10(p-value)
1	GO:0007169	Transmembrane receptor protein tyrosine kinase signaling pathway	50	35.52
2	GO:0001817	Regulation of cytokine production	45	5.82
3	GO:0007264	Small GTPase mediated signal transduction	38	12.49
4	GO:0048583	Regulation of response to stimulus	33	14.21
5	GO:0033559	Unsaturated fatty acid metabolic process	20	15.91
6	GO:0019221	Cytokine-mediated signaling pathway	15	10.76

C

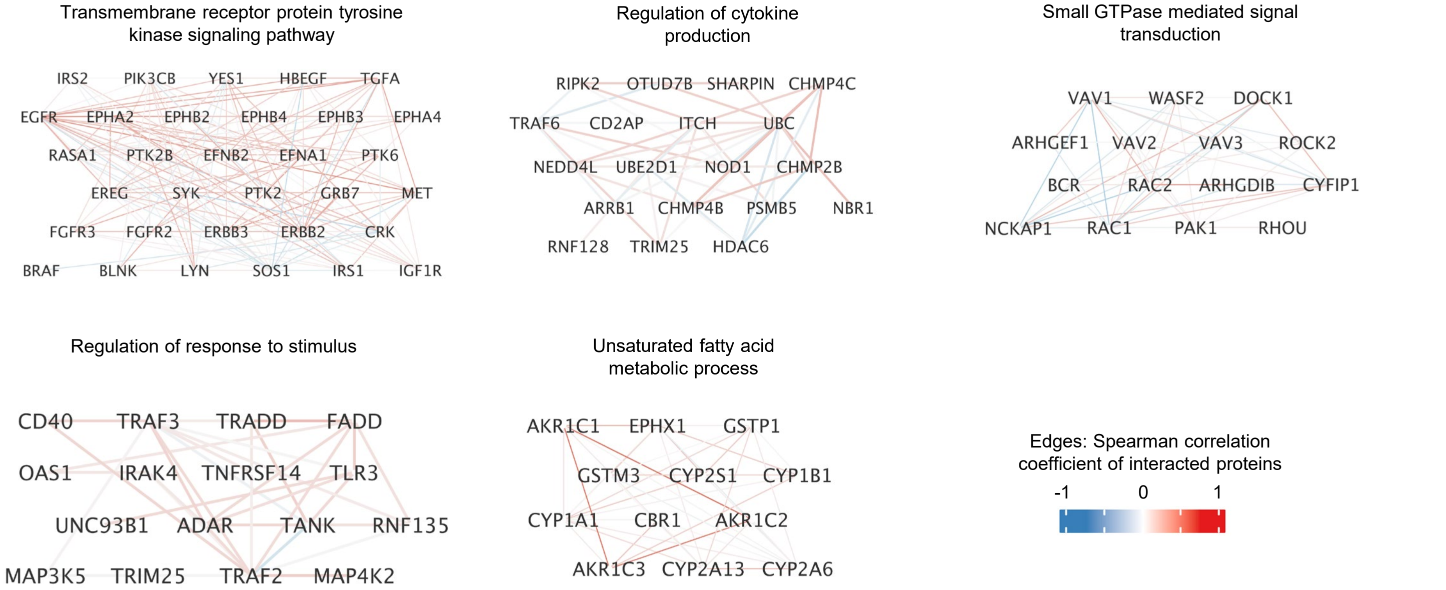

Figure S7. The gene network of subGO cluster 2.
(A) The interaction network of subGO cluster 2.
(B) The table showing the most enriched biological process in identified subclusters. The enrichment *P*-values and the total number of genes in each subcluster are shown.
(C) The interaction network showing example genes in indicated subclusters.

Figure S8

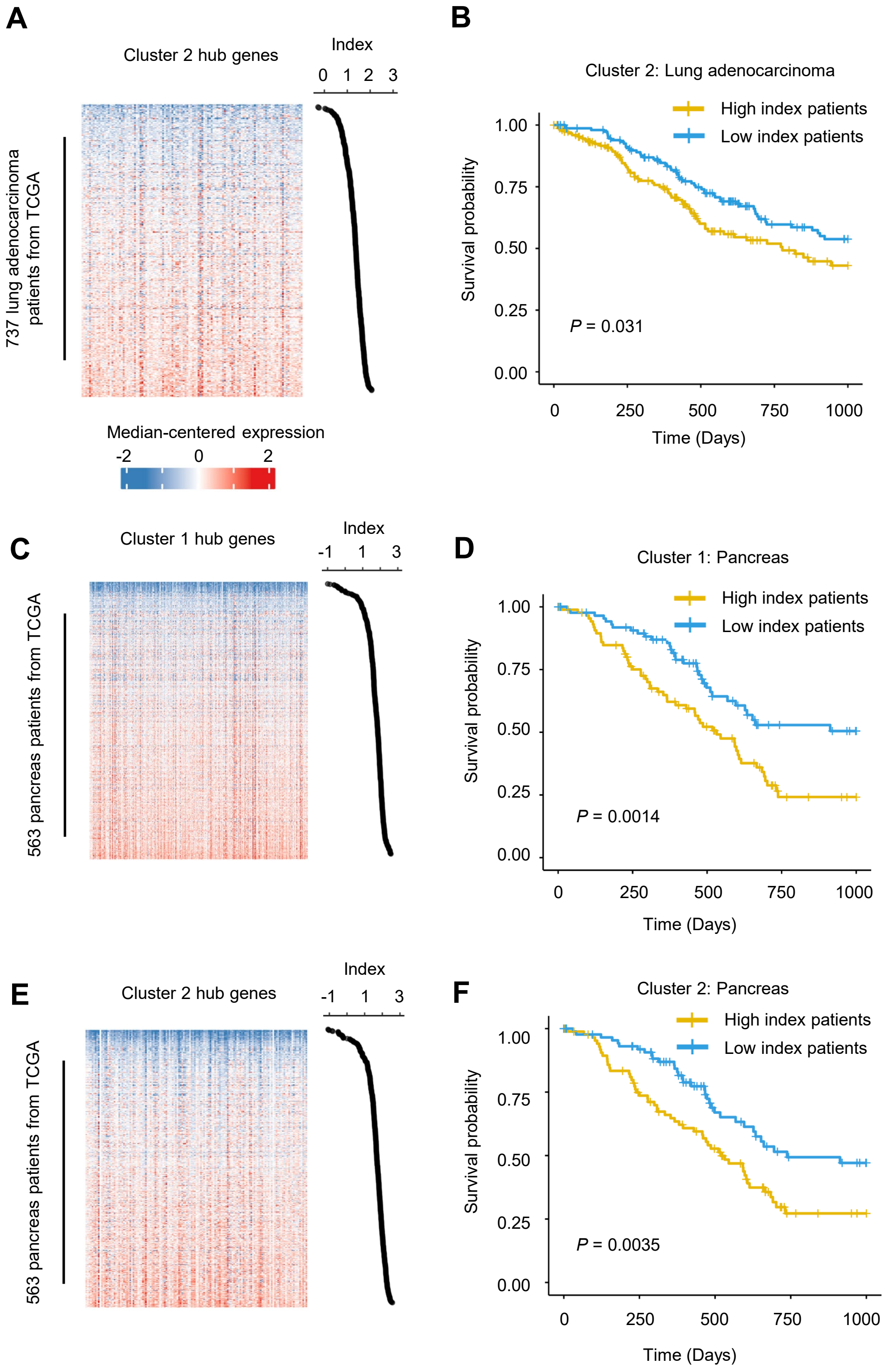

Figure S8. The expression of gene networks in cancer patients.
(A) Heatmap showing the relative expression of hub genes in cluster 2 across lung adenocarcinoma patients from TCGA.
(B) The Kaplan–Meier estimator shows the lung adenocarcinoma patients with vs. low cluster 2 network expression. The log-rank test *P*-value is shown.
(C) Heatmap showing the relative expression of hub genes in cluster 1 across pancreatic cancer patients from TCGA.
(D) The Kaplan–Meier estimator shows the pancreatic cancer patients with high vs. low cluster 1 network expression. The log-rank test *P*-value is shown.
(E) Heatmap showing the relative expression of hub genes in cluster 2 across pancreatic cancer patients from TCGA.
(F) The Kaplan–Meier estimator shows the pancreatic cancer patients with high vs. low cluster 2 network expression. The log-rank test *P*-value is shown.

Figure S9

A

Subclusters in cluster 3	GO ID	GO name	Gene number	-log10(p-value)
1	GO:0008380	RNA splicing	143	204.17
2	GO:0032543	Mitochondrial translation	95	159.36
3	GO:0042254	Ribosome biogenesis	84	119.59
4	GO:0002181	Cytoplasmic translation	70	82.30
5	GO:0000502	Proteasome complex	63	55.87
6	GO:0006119	Oxidative phosphorylation	37	71.58
7	GO:0006351	Transcription, DNA-templated	23	21.77

B

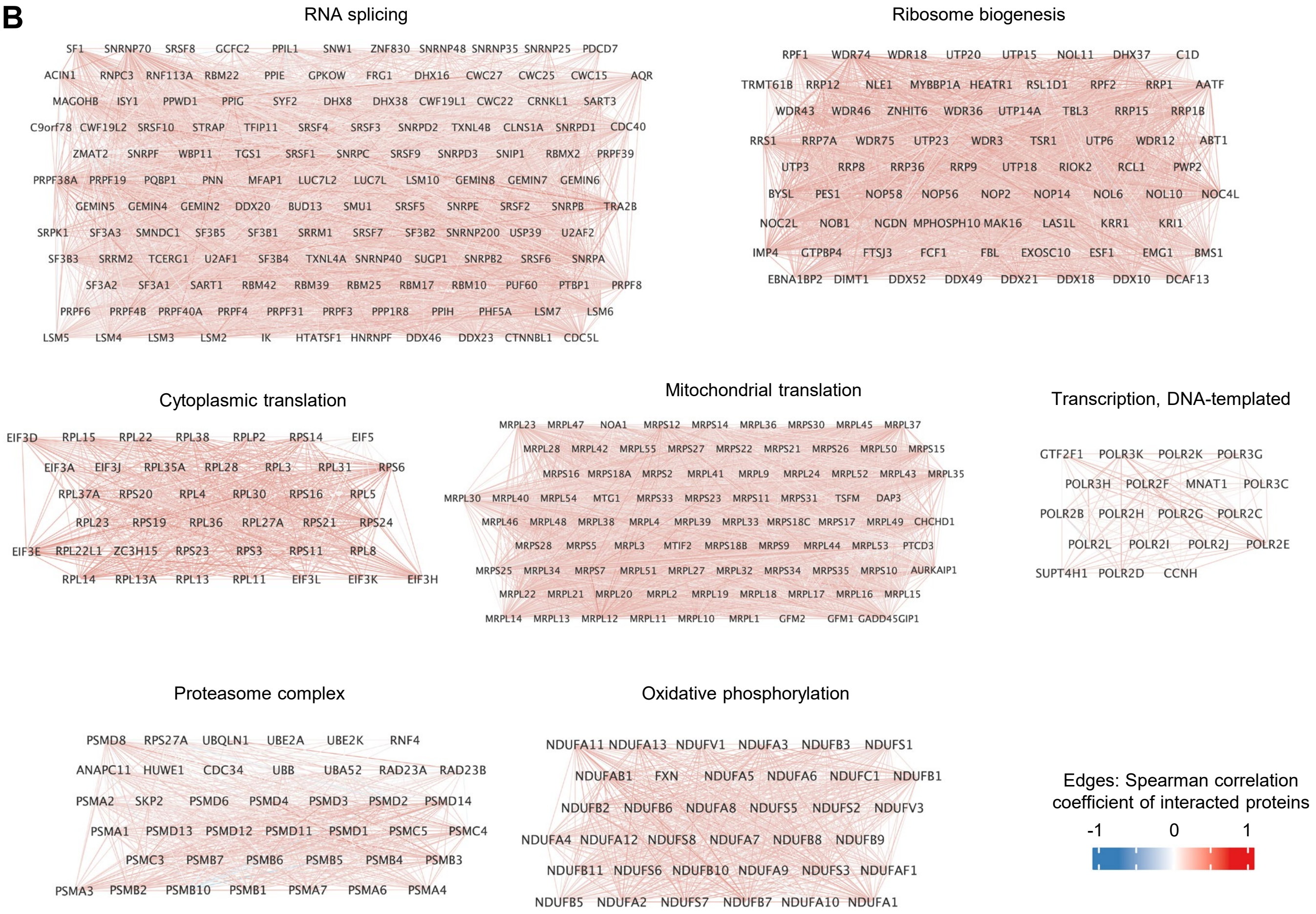

Figure S9. The gene network of subGO cluster 3.

(A) The table showing the most enriched biological process in identified subclusters. The enrichment *P*-values and the total number of genes in each subcluster are shown.

(B) The interaction network showing example genes in indicated subclusters.

Figure S10

A

Subclusters in cluster 4	GO ID	GO name	Gene number	-log10(p-value)
1	GO:0007049	Cell cycle	96	89.18
2	GO:0006508	Proteolysis	65	34.43
3	GO:0006260	DNA replication	54	59.19
4	GO:0006281	DNA repair	53	70.33
5	GO:0006325	Chromatin organization	33	32.44

B

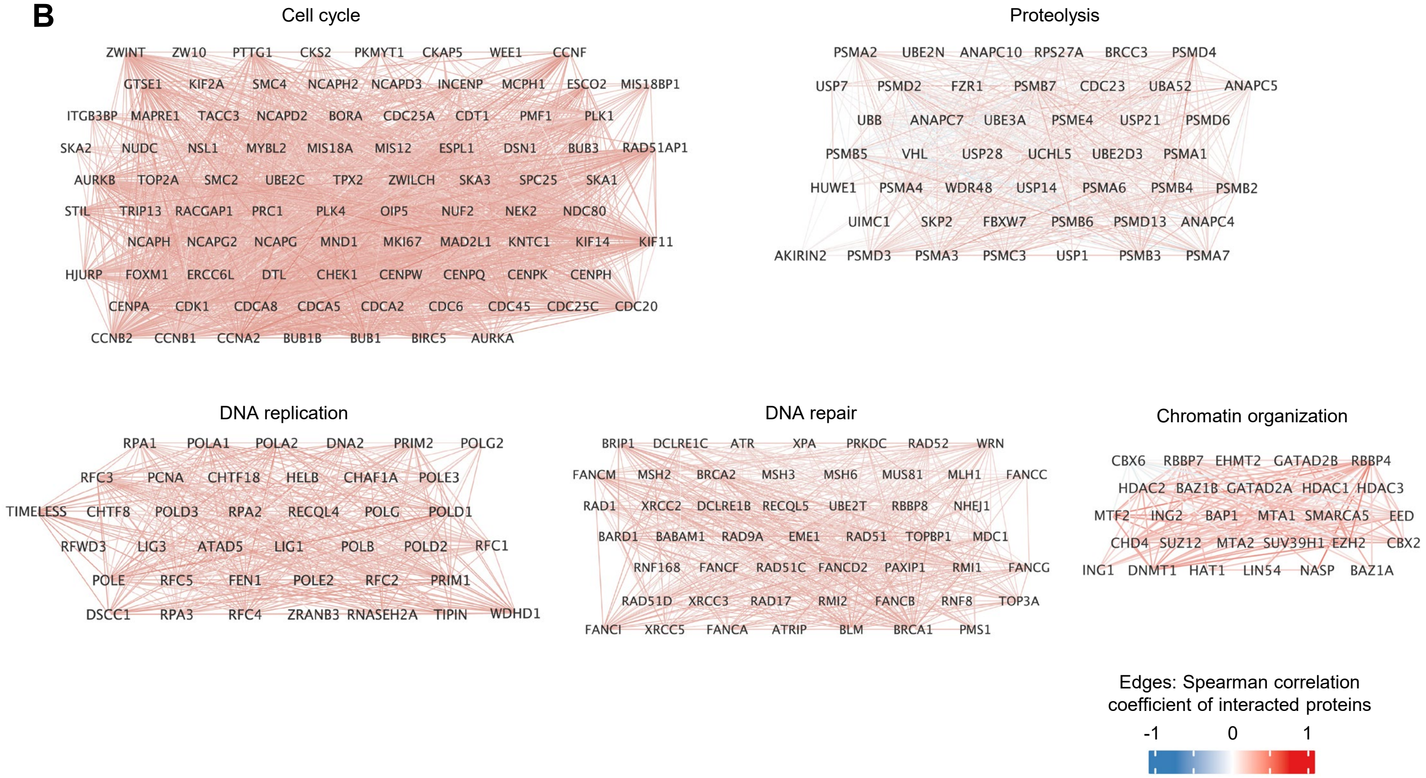

Figure S10. The gene network of subGO cluster 4.
(A) The table showing the most enriched biological process in identified subclusters. The enrichment *P*-values and the total number of genes in each subcluster are shown.
(B) The interaction network showing example genes in indicated subclusters.

Figure S11

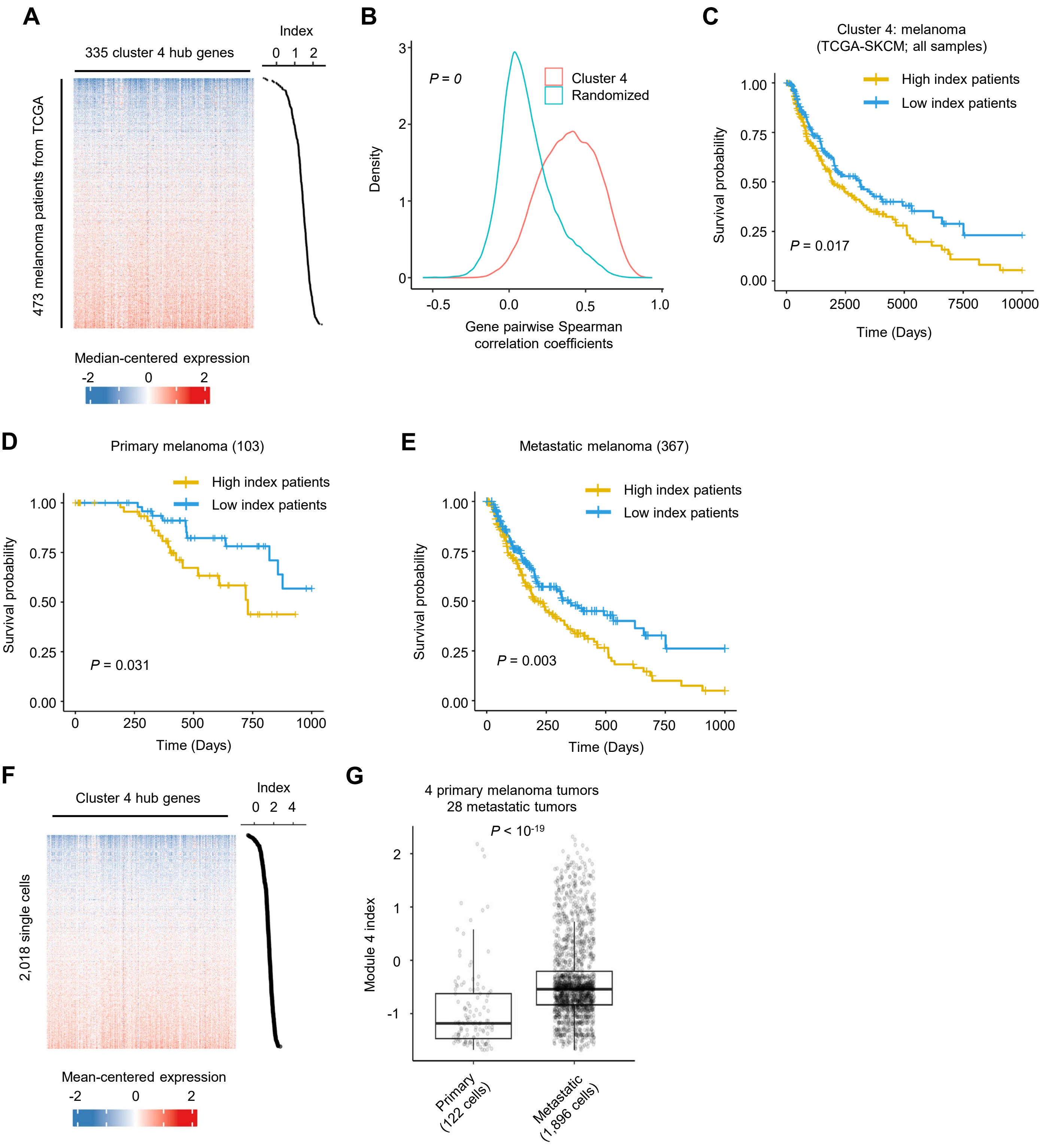

Figure S11. The conservation of module cluster 4 in melanoma patients.
(A) Heatmap showing the relative expression of hub genes in cluster 4 across melanoma patients from TCGA.
(B) The correlation of gene pairs from cluster 4 and random pairs. The Wilcoxon rank-sum test *P*-value is shown.
(C) The Kaplan–Meier estimator shows the melanoma patients with high (top 50%) vs. low (low 50%) cluster 4 network expression. The log-rank test *P*-value is shown.
(D) As in (C), the analyses using the data from the primary melanoma patients only are shown.
(E) As in (C), the analyses using the data from the metastatic melanoma patients only are shown.
(F) Heatmap showing the relative expression of cluster 4 hub genes across 2,018 single cells from melanoma cells. We calculated the module gene expression as the index value for each cell.
(G) Boxplot comparing the expression of cluster 4 gene module across single cells from primary and metastatic melanoma tumors. The Wilcoxon rank-sum test *P*-value is shown.

Figure S12

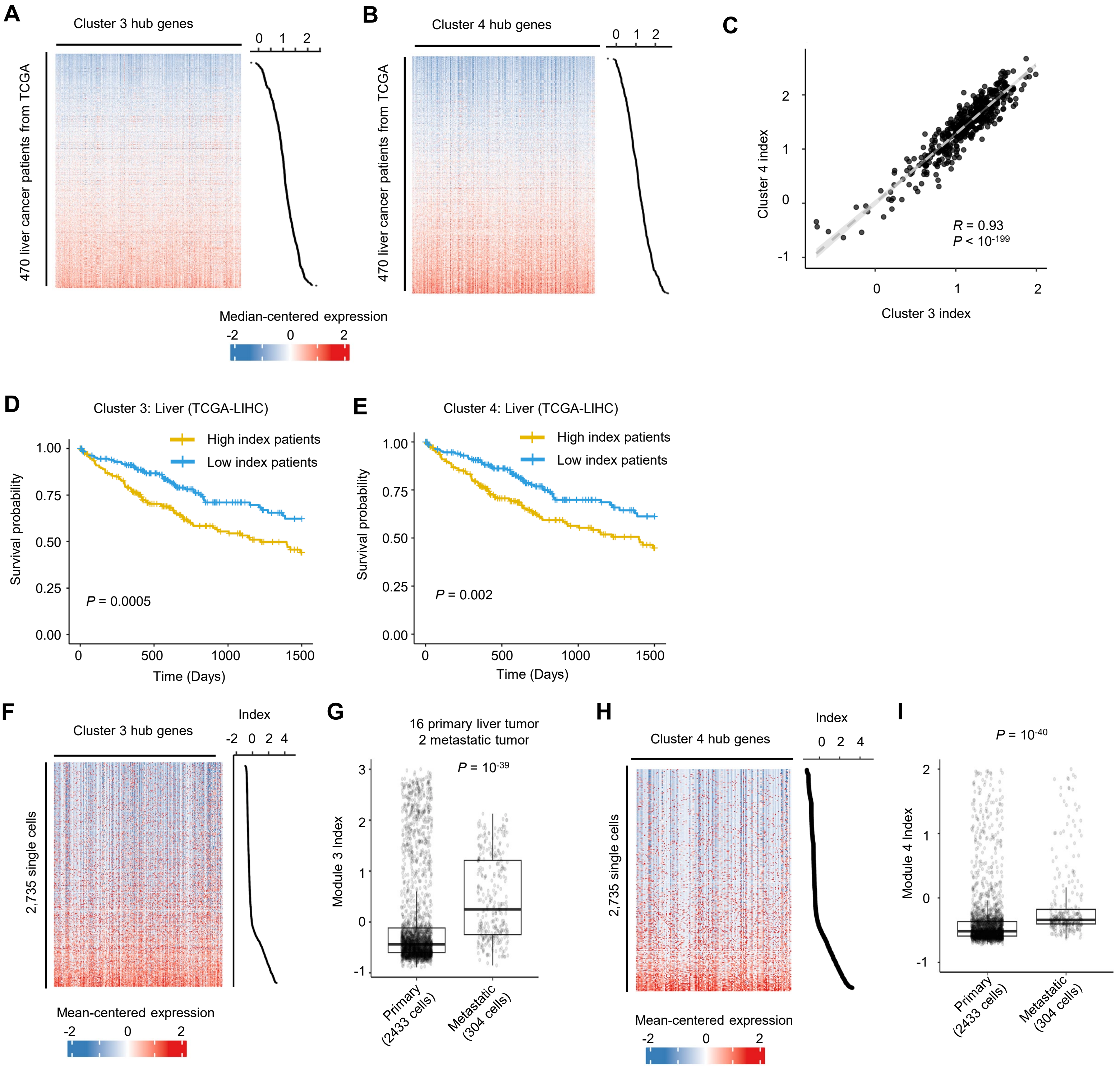

Figure S12. The conservation of module clusters 3 and 4 in liver cancer patients.

(A-B) Heatmap showing the relative expression of hub genes in cluster 3 (A) and cluster 4 (B) across liver cancer patients from TCGA.

(C) The correlation between cluster 3 and cluster 4 gene expression.

(D-E) The Kaplan–Meier estimator shows the liver cancer patients with high (top 50%) vs. low (low 50%) cluster 3 (D) and cluster 4 (E) network expression. The log-rank test *P*-value is shown.

(F) Heatmap showing the relative expression of cluster 3 hub genes across 2,735 single cells from liver tumors. We calculated the module gene expression as the index value for each cell.

(G) Boxplot comparing the expression of cluster 3 gene module across single cells from primary and metastatic liver tumors. The Wilcoxon rank-sum test *P*-value is shown.

(H) As in (F), the heatmap showing the expression of cluster 4 hub genes.

(I) As in (G), boxplot examing the expression of cluster 4 gene modules.

Figure S13

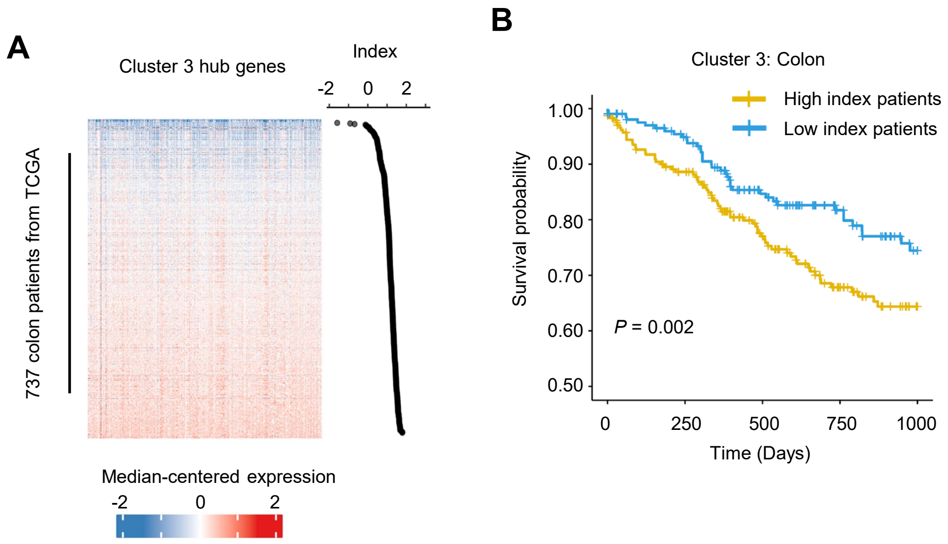

Figure S13. The expression of cluster 3 network in colon cancer patients.
(A) Heatmap showing the relative expression of hub genes in cluster 3 across colon cancer patients from TCGA.
(B) The Kaplan–Meier estimator shows the lung adenocarcinoma patients with vs. low cluster 3 network expression. The log-rank test *P*-value is shown.

Figure S14

A

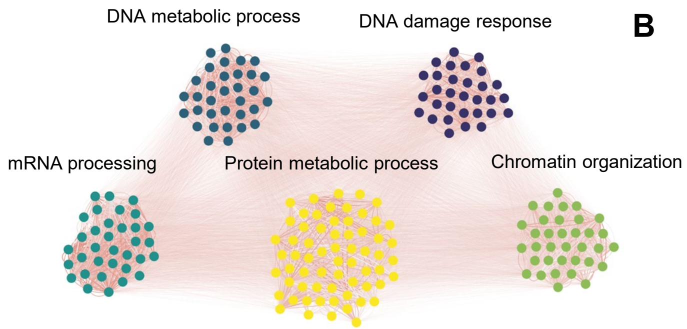

B

Subclusters in cluster 5	GO ID	GO name	Gene number	-log10(p-value)
1	GO:0019538	Protein metabolic process	64	25.67
2	GO:0006325	Chromatin organization	35	39.24
3	GO:0006397	mRNA processing	34	39.85
4	GO:0006259	DNA metabolic process	33	41.7
5	GO:0006974	DNA damage response	31	32.4

C

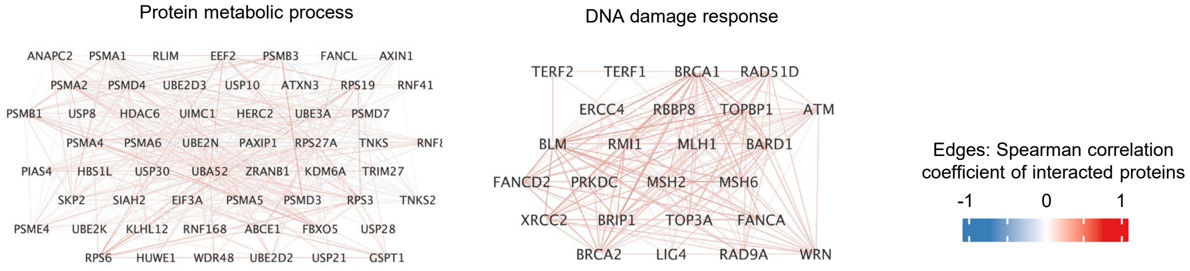

Figure S14. The gene network of subGO cluster 5.
(A) The interaction network of subGO cluster 5.
(B) The table showing the most enriched biological process in identified subclusters. The enrichment *P*-values and the total number of genes in each subcluster are shown.
(C) The interaction network showing example genes in indicated subclusters.

Figure S15

A

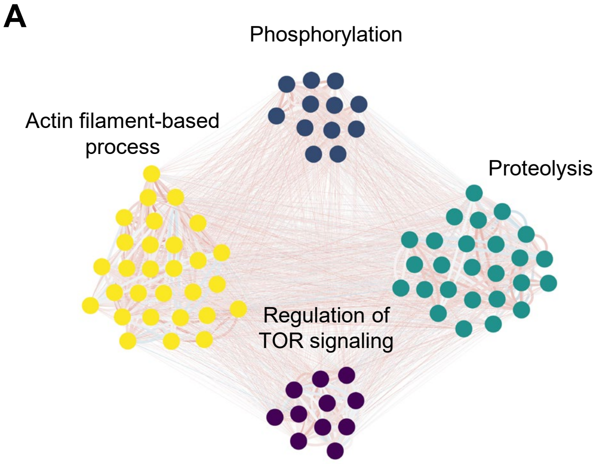

B

Subclusters in cluster 6	GO ID	GO name	Gene number	-log10(p-value)
1	GO:0030029	Actin filament-based process	29	13.81
2	GO:0006508	Proteolysis	26	5.05
3	GO:0016310	Phosphorylation	12	2.85
4	GO:0032006	Regulation of TOR signaling	11	20.42

C

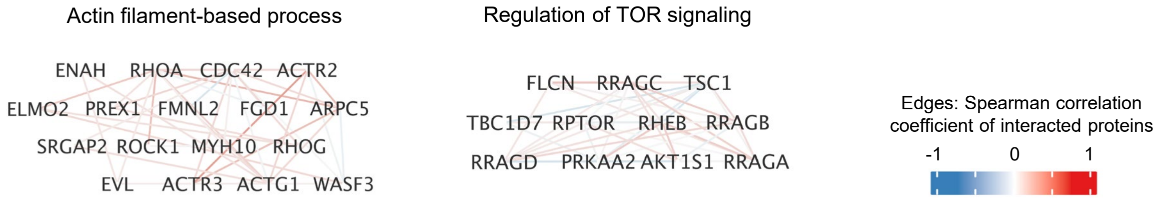

Figure S15. The gene network of subGO cluster 6.

(A) The interaction network of subGO cluster 6.

(B) The table showing the most enriched biological process in identified subclusters. The enrichment *P*-values and the total number of genes in each subcluster are shown.

(C) The interaction network showing example genes in indicated subclusters.

Figure S16

A

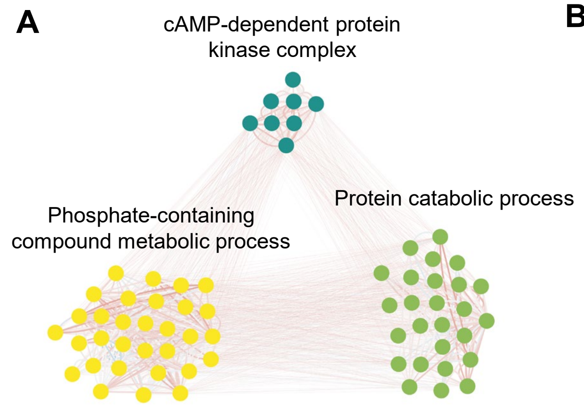

B

Subclusters in cluster 7	GO ID	GO name	Gene number	-log10(p-value)
1	GO:0006796	Phosphate-containing compound metabolic process	31	18.96
2	GO:0030163	protein catabolic process	26	6.00
3	GO:0005952	cAMP-dependent protein kinase complex	8	4.59

C

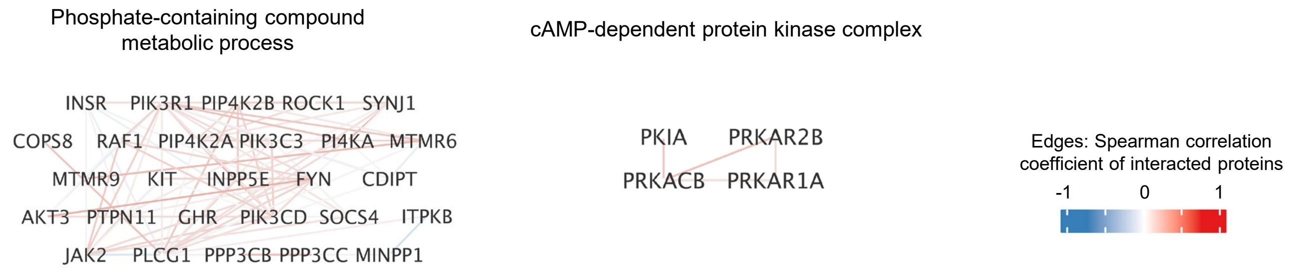

Figure S16. The gene network of subGO cluster 7.
(A) The interaction network of subGO cluster 7.
(B) The table showing the most enriched biological process in identified subclusters. The enrichment *P*-values and the total number of genes in each subcluster are shown.
(C) The interaction network showing example genes in indicated subclusters.

Figure S17

A

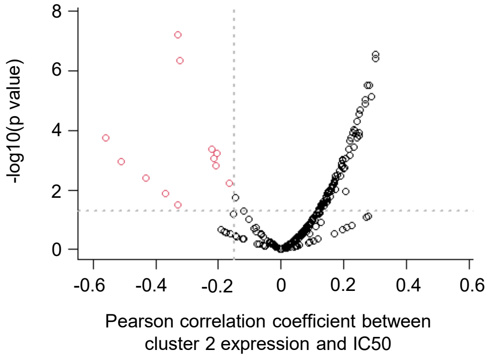

B

Drug	Target	Correlation	P-value
Lapatinib	EGFR, ERBB2	-0.559	1.77E-04
Afatinib (rescreen)	EGFR, ERBB2	-0.330	5.87E-08
Afatinib	EGFR, ERBB2	-0.323	4.44E-07
Cetuximab	EGFR	-0.222	4.17E-04
Gefitinib	EGFR	-0.216	8.97E-04
LY317615	PKCB	-0.206	5.71E-04

C

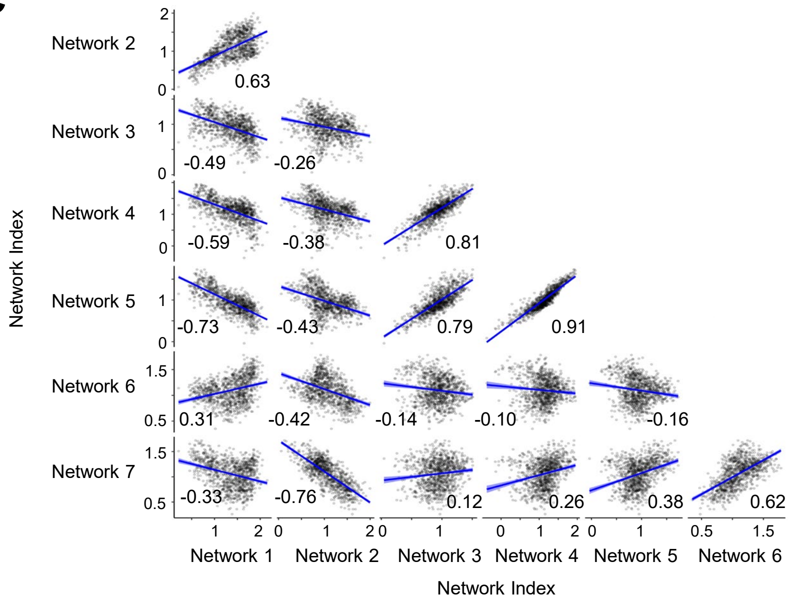

Figure S17. The correlation of drug response and cluster 2 gene expression.
(A) Scatter plot showing the Pearson correlation between IC50 and cluster 2 network expression vs. the -log10(*P*-value) from the regression test.
(B) The example drugs whose responses are most correlated with higher expression of cluster 2 network expression.
(C) The correlation between the expression of different gene networks across CCLE cell lines. The Pearson correlation coefficient values are shown.

SUPPLEMENTARY TABLES

Table S1. The statistics of cell lines from different lineage types analyzed by the MetMap project. For each lineage type, we showed the numbers and names of metastatic and nonmetastatic cell lines. And the fold-change cutoffs to select genes showing upregulation in metastatic cells are shown.

Table S2. The GO analyses of genes upregulated in metastatic cell lines. For each GO term and lineage type, we showed the enrichment score -log10(*P*-value), the number of genes, and the fractions of the genes overlapping with two out of the 28 selected GO terms and all GOs.

Table S3. The coexpressed subGO modules identified in this study.

Table S4. The overlap, Jaccard index, and Sørensen–Dice coefficient comparing the subGO modules and GO annotated child terms.

Table S5. The correlation between the subGO module expression and metastatic potentials across lineage types in Figure 3A.

Table S6. The performance of random forest regression models across different metastatic routes. The Pearson correlation coefficient values between the predicted and observed values, as well as *P*-values are shown.

Table S7. Node and edge features of network 1-7 from STRING analyses.

Table S8. The drug response indicated by the Pearson correlation coefficient values and regression *P*-values between the IC50 values and the network expression.
